# A Density Functional Theory and Semiempirical Framework for Trajectory Surface Hopping on Extended Systems

**DOI:** 10.1021/acs.jctc.5c01082

**Published:** 2025-10-17

**Authors:** Jan-Robert Vogt, Michael Schulz, Rafael Souza Mattos, Mario Barbatti, Maurizio Persico, Giovanni Granucci, Jürg Hutter, Anna Hehn

**Affiliations:** † 9179 Christian-Albrechts-University Kiel, Max-Eyth-Strasse 1, 24118 Kiel, Germany; ‡ 128791 Aix Marseille University, CNRS, ICR, 13397 Marseille, France; § Institut Universitaire de France, 75231 Paris, France; ∥ Dipartimento di Chimica e Chimica Industriale, Via Moruzzi 13, 56124 Pisa, Italy; ⊥ Department of Chemistry, 27217 University of Zurich, Winterthurerstrasse 190, 8057 Zurich, Switzerland; # Kiel Nano, Surface and Interface Science, 24118 Kiel, Germany

## Abstract

Nonadiabatic molecular dynamics simulations provide a theoretical understanding of various excited-state processes in photochemistry, offering access to band widths, radiative or nonradiative relaxation and corresponding lifetimes, excited-state energies, and charge transfer. The range of method developments within the framework of time-dependent density functional theory is exceedingly large for molecular quantum chemistry. Still, it shrinks significantly when aiming to treat periodic boundary conditions. To address this gap and complement existing software packages for solid-state nonadiabatic molecular dynamics, we present an interface between the CP2K electronic structure and the NEWTON-X surface hopping codes. The interface features the generation of initial conditions, as well as adiabatic and nonadiabatic molecular dynamics, based on phenomenological or numerical time-derivative couplings. Setups are validated on gas-phase pyrazine, with electronic absorption spectra and excited-state populations for transitions between the lowest singlet states being in agreement with established molecular quantum chemistry methods. Extending the system size to crystalline pyrazine, limitations of approximate couplings are discussed, and the efficiency and applicability of the interface are demonstrated by computing broad spectra over several eV and 100 fs trajectories, considering couplings between all 80th lowest excited states, at low computational cost with a mixed semiempirical density functional theory setup.

## Graphical abstract

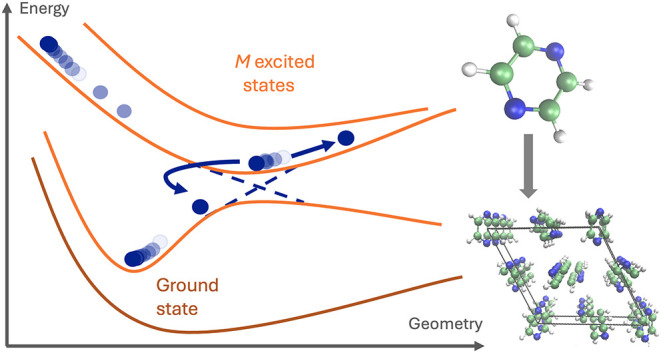

## 1 Introduction

The time-dependent study of photochemical properties in extended systems implying periodic boundary conditions, i.e., in solids, surfaces, or condensed-phase systems, requires a setup suitable for efficient nonadiabatic dynamics. Localization of the excitation manifold, ideally in combination with a hybrid quantum mechanics/molecular mechanics setup, can reduce computational timings and enable applications at a large scale.^1,2^ In combination, trajectory surface hopping (TSH) represents a powerful tool to avoid the scanning of complete potential energy surfaces: Among the mixed quantum-classical approaches, TSH restricts the classical description of nuclear motion to sampling a single trajectory and thus enabling straightforward parallelization.^3–9^ In general, internal consistency of trajectory surface hopping methods can be restored by adding decoherence corrections, then yielding satisfactory results in agreement with quantum wave packet computations.^10^ The advantage of TSH becomes even more significant when modeling systems with periodic boundary conditions (PBC). In contrast to modeling distinct molecules, the number of relevant excited states is usually much larger for extended system sizes.^11^ In these cases, TSH solely requires the computation of nuclear forces for the single currently populated state, while Ehrenfest methods, e.g., refer to forces averaged across all states.^8^

Especially for molecular systems, the success of TSH can moreover be reasoned with further method developments, e.g., for the efficient computation of nonadiabatic couplings (NACs): Explicit analytical differentiation^12–15^ is computationally demanding, and accurate integration of analytical couplings is still hampered due to the nevertheless required discretization in time.^16,17^ Time derivative couplings^17–19^ have therefore been suggested, relying on the numerical derivative of overlaps of wave functions of consecutive time steps. While the determinant derivative (DD) ansatz^20,21^ refers to overlaps of Slater determinants, the orbital derivative (OD) ansatz restricts the contributions to overlap matrices of Kohn–Sham orbitals, reducing computation times by at least 2 orders of magnitude while showing similar accuracies as DD.^22–25^ OD requires tracking of the phase and energetic ordering of the underlying Kohn–Sham orbitals, and it has therefore been suggested to rely on unitary-transformed instead of canonical orbitals.^26^ Furthermore, a modified OD approach applying an additional transformation step based on singular value decomposition has been developed to enhance numerical stability.^26,27^

Next to the mentioned ansätze for numerical time derivative couplings, local diabatization (LD) has been established as a standard approach for high stability in highly peaked nonadiabatic interactions, relying on overlap matrices computed within the DD or OD ansatz.^28,29^ Furthermore, the most cost-efficient alternative for computing NACs is given by phenomenological couplings, as defined, e.g., within the time-dependent Baeck-An (TDBA) approximation, relying solely on energy gaps and their second time derivatives.^30–33^ For ethylene and fulvene, it was shown that TDBA couplings provide a qualitatively correct description of the dynamics in comparison to analytical couplings, failing, however, in regions of strong nonadiabatic coupling and small velocities. More explicitly, adiabatic state populations and corresponding lifetimes are reproduced within the error bars of comparable results corresponding to exact couplings. The magnitudes of couplings as well as the corresponding order of coupling magnitudes for different pairs of states are, in general, well-reproduced with the trend to overestimate small-magnitude and underestimate large-magnitude couplings.

While the mentioned developments have significantly advanced TSH schemes in recent years, implementations and applications are often still restricted to molecular systems. Several trajectory surface hopping codes for periodic boundary conditions exist, including Pyxaid,^34^ Libra,^35–37^ Hefei,^38^ CPMD,^39–41^ ZAGREB,^42^ or GTSH,^43^ enabling nonadiabatic molecular dynamic simulations through interfaces with state-of-the-art second-party electronic structure programs. However, many of these rely on the neglect of back reaction (NBRA) approximation or are restricted in terms of coupling schemes and modularity. NBRA still represents a common standard approximation, assuming that ground-state nuclear forces can be used to propagate the nuclei, approximating electronic excitation energies as simple Kohn–Sham energy gaps and time-derivative couplings as derivatives of those Kohn–Sham orbitals.^44,45^ The NBRA increases efficiency and is considered a reliable approximation in cases where electronic excitation is not associated with significant structural changes, which is often well justified for slow nonadiabatic processes, such as electron–hole recombination. The approximation is, however, inadequate in cases where coupling between nuclear and electronic structure needs to be taken into account, as has been reasoned, e.g., for an accurate modeling of charge transfer.^46^ Furthermore, the mentioned interfaces for solid-state chemistry differ first of all in the underlying quantum-chemical electronic-structure program, providing thus only the corresponding characteristic features of these electronic-structure codes. Pyxaid and Hefei-NAMD are currently interfaced with Quantum Espresso and VASP, respectively, relying thus on a global plane wave basis for the electronic-structure computation. CPMD comprises its own surface hopping module, which is equally restricted to plane waves. Regarding the underlying electronic-structure code, the closest comparison to the interface presented in this contribution is possible with the Libra, ZAGREB, and GTSH interfaces, which enable all computations using the CP2K program package. In contrast to our contribution, the Libra-CP2K interface described in ref 47 employs the NBRA and calculates numerical time derivative couplings based on spin-adapted configurations, which represent Slater determinants for closed-shell structures. Combining CP2K with the Zagreb Surface Hopping NAMD code furthermore provides a setup for performing ΔSCF trajectory surface hopping, relying on Landau–Zener hopping probabilities.^42^ Finally, very recent work on the GTSH program package overcomes the NBRA, but is still restricted to OD couplings and assesses solely semiempirical GFN1-xTB-sTDA kernels for simulating photoinduced dynamics in carbon nanotubes.^43^

To address these limitations, we present a flexible and efficient interface between the surface hopping package NEWTON-X^48–50^ and the electronic structure code CP2K,^51^ supporting periodic systems and multiple nonadiabatic coupling approaches. The electronic-structure setup relies on the mixed Gaussian and plane waves (GPW) framework^51–53^ of CP2K (Section 2.1), which features both approximate hybrid density functional as well as semiempirical kernels for Sternheimer formulations within the Tamm-Dancoff approximation (TDA) of time-dependent density functional theory (TDDFT).^54–58^ NEWTON-X, in return, provides several choices for coupling constants (Section 2.2), including local diabatization,^28,29^ OD^23^ and time-dependent Baeck-An couplings.^32^ The interface is assessed by comparing excited-state (ES) dynamics for molecular pyrazine with analogous simulations using the already established NEWTON-X interface with the molecular quantum chemistry program package TURBOMOLE^59,60^ (Section 3.1). Applicability and efficiency of the mixed density functional theory and semiempirical setup is furthermore assessed for crystalline pyrazine (Section 3.2).

## 2 Methods

The underlying electronic-structure and surface hopping methods of the program packages CP2K^51,53–58^ and NEWTON-X^30,48–50^ are well documented, and the following summary of computational models is thus restricted to summarizing the structure and versatility of the interface, as well as to provide explicit formulas for the computation of numerical orbital derivative couplings.

### 2.1 Approximate Hybrid Density Functional Accuracy for Adiabatic Wave Functions, Potential Energies, and Excited-State Forces

Using the Born–Oppenheimer definition of adiabatic states, the total electronic wave function of the system Ψ­(**R**(*t*)) can be described as a linear expansion of such adiabatic eigenfunctions ψ^*M*^(**R**(*t*)) of all electronic states *M* with corresponding expansion coefficients *d* ^*M*^(*t*)­

1 $$\left|\Psi \left(R\left(t\right)\right)\right\rangle =\sum_{M}d^{M}\left(t\right)\left|\psi^{M}\left(R\left(t\right)\right)\right\rangle$$

depending on the nuclear coordinates **R** of time step *t*. The time propagation of coefficients *d* ^*M*^(*t*) is hereby determined by the time-dependent Schrödinger equation

2 $$i\frac{dd^{M}\left(t\right)}{dt}=\sum_{N}d^{N}\left(t\right)\left[\delta_{\mathrm{MN}}E_{N}\left(R\left(t\right)\right)-i\tau_{\mathrm{MN}}\left(t\right)\right]$$

referring to adiabatic potential energy surfaces *E*_*M*_(**R**(*t*)) and nonadiabatic coupling elements (NACs) τ_*MN*_(*t*)­

3 $$\tau_{\mathrm{MN}}\left(t\right)=\left\langle \psi^{M}\left(R\left(t\right)\right)\right|\frac{\partial }{\partial t}\psi^{N}\left(R\left(t\right)\right)\rangle$$

The adiabatic excited-state wave functions |ψ^*M*^(**R**(*t*))⟩ are not directly accessible within a time-dependent density functional theory framework, but can be approximated, implying a configuration interaction ansatz with singly excited Slater determinants |Φ_*aiσ*_⟩

4 $$\left|\psi^{M}\right\rangle =\sum_{\mathrm{ia}\sigma }c_{\mathrm{ai}\sigma }^{M}\left|\Phi_{\mathrm{ai}\sigma }\right\rangle$$

The singly excited determinants |Φ_*aiσ*_⟩ describe an excitation from the occupied to the virtual molecular orbital space, indicated by indices {*i*, *j*,...} and {*a*, *b*,...}, respectively. σ denotes the electronic spin index. Within the Tamm-Dancoff approximation (TDA), the expansion coefficients **c** ^*M*^ are defined based on the excited-state TDA eigenvectors **X** ^*M*^ ^26,61^

5 $$c_{\mathrm{ai}\sigma }^{M}=X_{\mathrm{ai}\sigma }^{M}$$

with the latter stemming from the Sternheimer formulation of the hermitian TDA eigenvalue problem

6 $${\mathrm{AX}}^{M}=\Omega_{M}{\mathrm{SX}}^{M}$$

7 $$\sum_{\kappa k}\left[F_{\mu \kappa \sigma }\delta_{\mathrm{ik}}-F_{\mathrm{ik}\sigma }S_{\mu \kappa }\right]X_{\kappa k\sigma }^{M}+\sum_{\kappa \lambda }Q_{\mu \kappa }^{T}K_{\kappa \lambda \sigma }\left[X^{M}\right]C_{\lambda i\sigma }=\sum_{\kappa }\Omega_{M}S_{\mu \kappa }X_{\kappa i\sigma }^{M}$$

with corresponding excitation energies Ω_*M*_ = *E*_*M*_ – *E*_0_. **S** represents the standard overlap matrix over atomic orbitals, {μ, ν,...}, and **Q** the projection operator onto the virtual orbital space inherent to the Sternheimer formulation. Within the latter, the virtual orbital space is omitted, thus the eigenvectors *X*_*aiσ*_^*M*^ of eq 5 are transformed according to standard transformation rules from the molecular orbital to the atomic orbital basis with the MO coefficients **C**. Depending on the chosen ground-state reference and excited-state kernel, the explicit Kohn–Sham **F** and kernel **K** matrix elements comprise different one- and two-electron contributions (see Figure 1); in the case of hybrid functionals, these would be one-electron, Coulomb, exact exchange, as well as functional-dependent exchange-correlation (XC) kernel contributions, with the computationally demanding exact exchange integrals being optionally approximated within the auxiliary density matrix method (ADMM).^57,62^ Next to a conventional hybrid density functional kernel, semiempirical kernels based on the simplified Tamm-Dancoff approximation (sTDA) reduce computational timings, parametrizing Coulomb and exchange integral contributions.^57^

**Figure 1:**
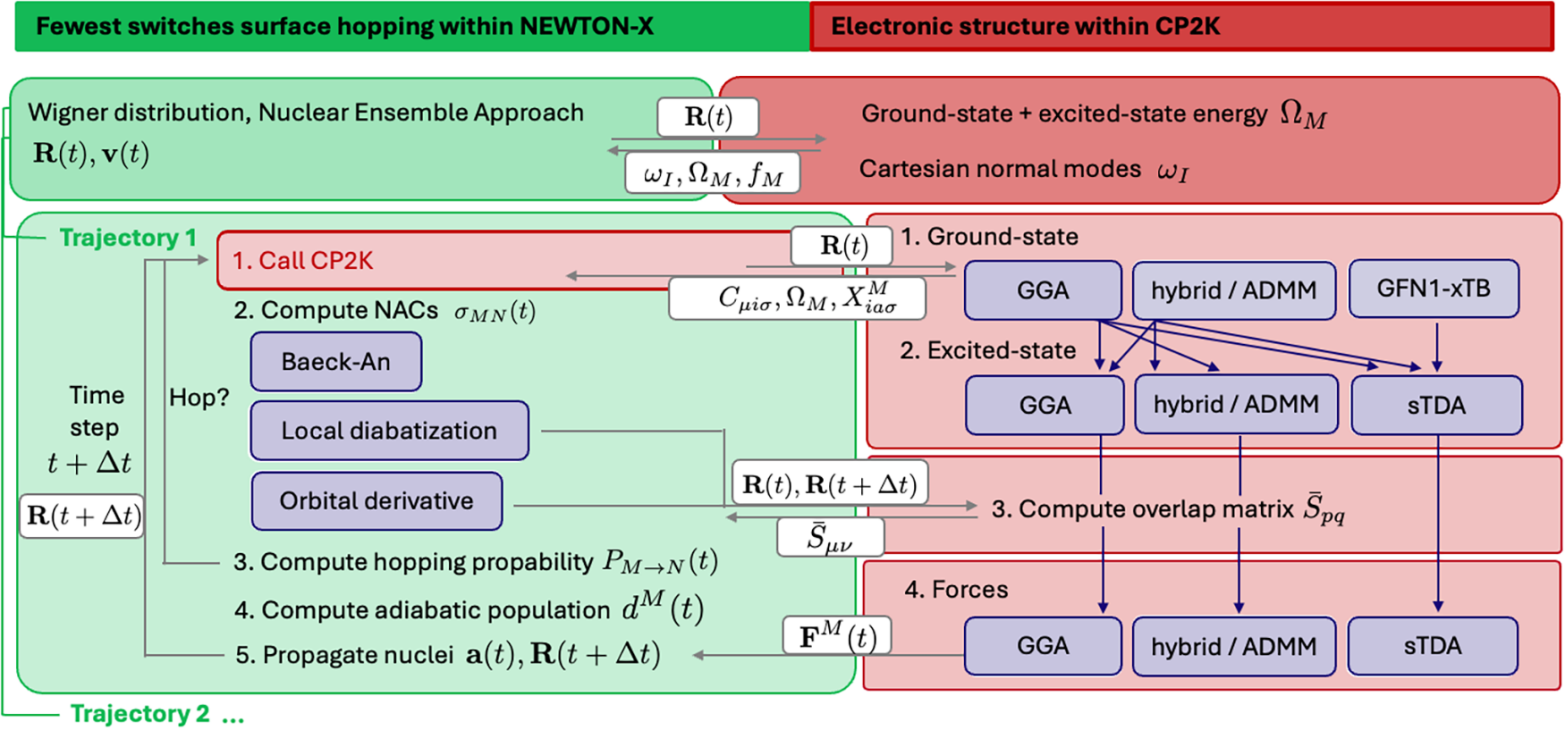
Interface of CP2K and NEWTON-X: NEWTON-X provides Cartesian coordinates of subsequent time steps **R**(*t*), **R**(*t* + Δ*t*) to CP2K, which in return passes molecular orbital coefficients **C**, Cartesian normal modes **ω**, excited-state eigenvalues **Ω** and amplitudes **X**, oscillator strengths **f**, electronic-state forces **F** and, depending on the choice for coupling constants, the overlap matrix **S̅** over wave functions of two subsequent time steps. Next to the general infrastructure, including e.g., adiabatic dynamics, thermostats, and decoherence corrections, NEWTON-X enables choosing between time-dependent Baeck-An or orbital derivative couplings or to rely on local diabatization. CP2K offers the modular choice to combine various ground-state references, e.g., density functionals within the generalized gradient approximation (GGA), hybrid functionals implying the auxiliary density matrix method (ADMM) or semiempirical GFN1-xTB references with corresponding excited-state kernels within the simplified or conventional Tamm-Dancoff approximation, the former abbreviated as sTDA.

We note that, within the TDA, hermiticity of the orbital-rotation matrix **A** ensures that the resulting CIS-type wave functions ψ^*M*^ are orthogonal to each other and that eq 5 is an adequate choice for expansion coefficients **c** ^*M*^. Orthogonality is, however, in general not trivial and, when expanding the formalism to full TDDFT, mutually orthogonal wave functions need to be constructed by relying on the hermitian formulation of the full TDDFT eigenvalue problem.^26^ Expansion coefficients **c** ^*M*^ are then given in terms of ground-state KS energy eigenvalues ε_*pσ*_ as^63^

8 $$c_{\mathrm{ia}\sigma }^{M}={\left(\varepsilon_{a\sigma }-\varepsilon_{i\sigma }\right)}^{-1/2}\left(X_{\mathrm{ia}\sigma }^{M}+Y_{\mathrm{ia}\sigma }^{M}\right)$$

with the TDDFT deexcitation amplitudes **Y** ^*M*^ being defined by solving the full TDDFT eigenvalue problem. Equation 8 thereby only holds for generalized gradient approximation (GGA) functionals, requiring further modifications for hybrid functionals, as explained in refs 63–65 Similar to eq 8, an alternative common choice for **c** ^*M*^ within TDA defines — in contrast to eq 5 — the coefficients in dependence of the excitation energy Ω_*M*_,^66^

9 $$c_{\mathrm{ia}\sigma }^{M}=\sqrt{\frac{\varepsilon_{i\sigma }-\varepsilon_{a\sigma }}{\Omega_{M}}}X_{\mathrm{ia}\sigma }^{M}$$

The following discussion will however always refer to eq 5.

Based on eq 7, ES nuclear gradients can be formulated by setting up a variational Lagrangian *L* ^*M*^ for the chosen excited state *M*, as outlined in ref 57. The force **F** ^*M*^, given as the negative derivative of the variational Lagrangian with respect to the nuclear coordinates **R** of nuclei α

10 $$F_{\alpha }^{M}\left(t\right)=-\nabla_{\alpha }L^{M}\left(R\left(t\right)\right)$$

determines, according to Newton’s first law of motion, the acceleration of each nuclei α.

### 2.2 Efficient Approximations for Nonadiabatic Couplings

Nonadiabatic coupling vectors (NACs) τ_*MN*_(*t*) describe the interaction between electronic states *M* and *N*. Within Tully’s fewest switches surface hopping,^18^ they are required for the computation of hopping probabilities. An analytical differentiation, defining NACs as the product of velocity **v** times the analytical derivative of the adiabatic state ψ^*N*^ with respect to the Cartesian nuclear coordinate

11 $$\tau_{\mathrm{MN}}\left(t\right)=\left\langle \psi^{M}\left(R\left(t\right)\right)\right|\frac{\partial }{\partial t}\psi^{N}\left(R\left(t\right)\right)\rangle =v\cdot \left\langle \psi^{M}\left(R\left(t\right)\right)\right|\nabla \left|\psi^{N}\left(R\left(t\right)\right)\right\rangle$$

however, requires significant implementation and computational effort. Moreover, since analytical NACs are available at time step *t* only, numerical differentiation, involving evaluation at two time steps *t* and *t* + Δ*t*, is often considered more robust. Several more cost-efficient approximations relying on the idea of numerical differentiation by Hammes-Schiffer and Tully^18^ have thus been established, approximating NACs with a 4-point formula according to

12 $$\tau_{\mathrm{MN}}\left(t\right)\approx \frac{1}{4\Delta t}\left[3{\mathrm{\tau ̅}}_{\mathrm{MN}}\left(t,t-\Delta t\right)-3{\mathrm{\tau ̅}}_{\mathrm{NM}}\left(t,t-\Delta t\right)-{\mathrm{\tau ̅}}_{\mathrm{MN}}\left(t-\Delta t,t-2\Delta t\right)+{\mathrm{\tau ̅}}_{\mathrm{NM}}\left(t-\Delta t,t-2\Delta t\right)\right]$$

τ̅_*MN*_(*t*, *t* – Δ*t*) can be obtained either by straight-forwardly evaluating the resulting overlap matrices of Slater determinants (Determinant derivative (DD) ansatz^20,21,24,25,49^ ) or by first applying the Slater–Condon rules to reduce resulting contributions to overlap matrices of Kohn–Sham molecular orbitals (Orbital derivative (OD) approach^22,23^ ). More explicitly, DD overlap couplings are based on the wave function ansatz of eq 5 given as

13 $${\mathrm{\tau ̅}}_{\mathrm{MN}}^{\mathrm{DD}}\left(t,t-\Delta t\right)=\sum_{\mathrm{iajb}\sigma }X_{\mathrm{ia}\sigma }^{M}\left(t\right)X_{\mathrm{jb}\sigma }^{N}\left(t+\Delta t\right)\left\langle \Phi_{\mathrm{ia}\sigma }\left(R\left(t\right)\right)\right|\Phi_{\mathrm{jb}\sigma }\left(R\left(t+\Delta t\right)\right)\rangle$$

implying the computation of overlap matrices over singly excited determinants |Φ_*iaσ*_(**R**(*t*))⟩. These overlap matrices can be evaluated by applying Löwdin’s rule, requiring the computation of all pairs of possible determinants |Φ_*iaσ*_(**R**(*t*))⟩ and |Φ_*jbσ*_(**R**(*t*))⟩. In contrast, the OD ansatz is obtained by first applying the Slater–Condon rules and subsequently evaluating the resulting overlap expressions

14 $${\mathrm{\tau ̅}}_{\mathrm{MN}}^{\mathrm{OD}}\left(t,t-\Delta t\right)=\sum_{\mathrm{ia}\sigma }{\left(X_{\mathrm{ai}\sigma }^{M}\left(t\right)\right)}^{T}X_{\mathrm{ai}\sigma }^{N}\left(t-\Delta t\right)+\sum_{\mathrm{iab}\sigma }{\left(X_{\mathrm{ai}\sigma }^{M}\left(t\right)\right)}^{T}X_{\mathrm{bi}\sigma }^{N}\left(t-\Delta t\right){\mathrm{S̅}}_{\mathrm{ab}\sigma }\left(t,t-\Delta t\right)-\sum_{\mathrm{ija}\sigma }{\left(X_{\mathrm{ai}\sigma }^{M}\left(t\right)\right)}^{T}X_{\mathrm{aj}\sigma }^{N}\left(t-\Delta t\right){\mathrm{S̅}}_{\mathrm{ji}\sigma }\left(t,t-\Delta t\right)$$

The computational advantage results from the fact that *S̅*_*pqσ*_(*t*, *t* – Δ*t*) represents an overlap matrix over Kohn–Sham orbitals |ϕ_*pσ*_(**R**(*t*))⟩

15 $${\mathrm{S̅}}_{\mathrm{pq}\sigma }\left(t,t-\Delta t\right)=\left\langle \phi_{p\sigma }\left(R\left(t\right)\right)\right|\phi_{q\sigma }\left(R\left(t-\Delta t\right)\right)\rangle$$

which is provided via the electronic-structure program. Further approximations have been suggested, with time-dependent Baeck-An couplings being a computationally efficient representative relying solely on electronic-state energies as input,^30^

$$\tau_{\mathrm{MN}}^{\mathrm{TDBA}}\left(t,t-\Delta t\right)=\{\begin{array}{cc} \frac{\mathrm{sgn}\left(\Delta E_{\mathrm{MN}}\right)}{2}\sqrt{\frac{1}{\Delta E_{\mathrm{MN}}}\frac{d^{2}\Delta E_{\mathrm{MN}}}{dt^{2}}} & \mathrm{if}\frac{1}{\Delta E_{\mathrm{MN}}}\frac{d^{2}\Delta E_{\mathrm{MN}}}{dt^{2}}>0\\ 0 & \mathrm{if}\frac{1}{\Delta E_{\mathrm{MN}}}\frac{d^{2}\Delta E_{\mathrm{MN}}}{dt^{2}}\leq 0 \end{array}$$ 16

with Δ*E*_*MN*_ = *E*_*M*_ – *E*_*N*_. Last but not least, couplings become negligible when relying on diabatic wave functions, constructed via local diabatization^29^ based on a transformation matrix that depends on the overlap matrix *S̅*_*pqσ*_(*t*, *t* – Δ*t*). Local diabatization interpolates between two time steps and thus avoids sampling of very peaked NACs, enabling the choice of larger time steps.

### 2.3 Interface Setup Including Initial Conditions and State and Phase Tracking

A graphical overview of the technical architecture of the CP2K-NEWTON-X interface is given in Figure 1, detailing how inputs and outputs are exchanged and visualizing the modular option to choose different combinations of ground-state references, implying corresponding Kohn–Sham matrix elements **F** and MO energies **ε**, as well as excited-state kernels, with corresponding excitation energies Ω_*M*_ and excitation amplitudes **X** ^*M*^. As visualized in Figure 1, these quantities are parsed from CP2K to NEWTON-X and enable the computation of matching coupling constants. Data exchange thereby depends on whether the NEWTON-X classical series (CS) or NEWTON-X new series (NS) is employed, with the former being based on Perl scripts enabling data exchange via text files, while the latter relies on monolithic Fortran code to maximize computational performance and data management, now enabling data exchange through memory.^50^ The interface is set up such that the complete general infrastructure of CP2K and NEWTON-X is made accessible, thus NEWTON-X is providing — next to the discussed features in the benchmark studies of this contribution — e.g., adiabatic dynamics, thermostats, and decoherence corrections. As outlined, choices for NACs comprise TDBA and OD couplings as well as local diabatization based on the latter. CP2K, in return, provides the modular choice to rely on various ground-state references, including density functionals within the generalized gradient approximation (GGA), hybrid functionals, also within the auxiliary density matrix method (ADMM), as well as semiempirical GFN1-xTB references.^67,68^ The thereon-based excited-state kernel can be chosen flexibly, thus enabling the combination of, e.g., GGA ground-state references with an ADMM-approximated hybrid functional excited-state kernel. Computational costs are furthermore reduced by 1 order of magnitude if relying on sTDA instead of the conventional TDA kernels.^57^ Semiempirical approximations can thus be introduced for both the ground-state reference (within GFN1-xTB) as well as for the excited-state kernel (within sTDA). We also refer to refs 51,69 and 48–50 for further information on the CP2K and NEWTON-X program packages.

Furthermore, various options to set up an ensemble of initial states are automatically provided by NEWTON-X.^50^ The nuclear ensemble approach, e.g., defines mass-scaled Cartesian coordinates and momenta based on a Wigner probability distribution *P*_W_

17 $$P_{W}\left(\mathrm{R̅},\mathrm{P̅}\right)=\Pi_{I=1}^{N_{F}}\frac{\alpha_{I}}{\pi \hbar }\exp\left(-\frac{\alpha_{I}}{\hbar \omega_{I}}\left(\omega_{I}^{2}{\mathrm{R̅}}_{I}^{2}+{\mathrm{P̅}}_{I}^{2}\right)\right)$$

with $\alpha_{I}=\tanh\left(\frac{\hbar \omega_{I}}{2k_{B}T}\right)$ , *R̅*_*I*_ = μ_*I*_^1/2^ *R*_*I*_, *P̅*_*I*_ = μ_*I*_^–1/2^ *P*_*I*_, the reduced mass μ_*I*_ and vibrational frequencies ω_*I*_. For systems implying periodic boundary conditions lacking rotational degrees of freedom, the thermal population of vibrational states thereby refers to the reduced number of degrees of freedom *N*_F_ = 3*N*_at_ – 3. Starting from the so-generated manifold of initial structures, propagated geometries and velocities, **R**(*t*) and **v**(*t*), are obtained according to the classical equations of motion using the velocity Verlet algorithm

18 $$R\left(t+\Delta t\right)=R\left(t\right)+v\left(t\right)\Delta t+\frac{1}{2}a\left(t\right)\Delta t^{2}$$

19 $$v\left(t+\Delta t\right)=v\left(t\right)+\frac{1}{2}\left(a\left(t\right)+a\left(t+\Delta t\right)\right)\Delta t$$

requiring the computation of acceleration **a**(*t*) as defined via eq 10 and thus the computation of both ES energies and ES nuclear gradients.

Furthermore, expansion coefficients *d*_*M*_(*t*) are propagated according to the time-dependent Schrödinger equation (eq 2) based on the computed NACs. The expansion coefficients *d*_*M*_(*t*) enable to propagate state occupations based on FSSH hopping probabilities *P*

20 PM→N(t)=max[0,−2Δt|dM(t)|2τNM(t)Re[dM(t)dN*(t)]]

i.e., to switch the state from *M* to *N* if the hopping probability fulfills ∑_*K* = 1_^*N*–1^ *P*_*M*→*K*_ < *r*_*t*_ ≤ ∑_*K* = 1_^*N*^ *P*_*M*→*K*_ with *r*_*t*_ representing a randomly chosen number drawn from a uniform distribution over the interval [0, 1]. To ensure correct time propagation of excited-state amplitudes and maintain continuity in state labeling, the interface enforces state tracking via overlap-based orthonormality checks between subsequent time steps

21 $$\sum_{\mathrm{ij}\mu \nu \sigma }X_{i\mu \sigma }^{N}\left(t\right)S_{\mu \nu }\left(t\right)X_{j\nu \sigma }^{M}\left(t-\Delta t\right)=\delta_{\mathrm{MN}}$$

with the excited-state eigenvectors **X** ^*M*^ being again expressed within the AO-based formulation of the Sternheimer equations. Moreover, phase tracking is ensured by comparing the phase of the orthonormalized excited-state eigenvectors along trajectories.

## 3 Results and Discussion

### 3.1 Validation for Molecular Pyrazine

Pyrazine represents an extensively studied molecule in photochemistry. The internal conversion from the second to the first bright excited state was investigated both experimentally with time-resolved photoelectron spectroscopy measurements, as well as theoretically using time-dependent density functional theory.^48,64,70–74^ The absorption spectrum of molecular pyrazine in the gas phase is thus characterized by two bands at energies of 3.8 and 4.8 eV, being classified as *n* → π* transition (HOMO →LUMO) to the first singlet state of ^1^ *B*_1u_ character and a second π → π* transition (HOMO–1 →LUMO) to the second excited singlet state of ^1^ *B*_2u_ symmetry. The third bright state corresponds to a π → π* transition (HOMO–1 →LUMO+1) with ^1^ *B*_3u_ character. To validate the implementation in comparison to the already interfaced molecular quantum chemistry code TURBOMOLE^59,60^ and to compare results with the extensive computational as well as experimental literature data, we computed the broadened absorption spectrum as well as the excited-state lifetimes for molecular pyrazine in the gas phase.

Computational setups for the molecule are chosen such that they enable a comparison with molecular quantum-chemistry codes, with all-electron computations being performed within the Gaussian and augmented plane wave framework (GAPW),^54,75^ relying on the B3LYP functional^76^ in combination with the def2-TZVP basis set of Ahlrichs^77,78^ and all-electron pseudo potentials.^75^ To further assess setups suitable for periodic boundary conditions, GAPW was replaced by the Gaussian and plane wave framework (GPW)^79^ and all-electron basis sets by valence-only MOLOPT basis sets^80^ in combination with corresponding pseudo potentials.^81^ Broadened absorption spectra were obtained using the nuclear ensemble approach, sampling over 500 initial geometries and velocities generated according to a Wigner probability distribution.^82^ For the simulated cross section, a Lorentzian line shape with a broadening of 0.05 eV was used. Data files relevant for the presented computational results are provided in ref 83 and on Materials Cloud.

The broadened absorption spectrum is shown in Figure 2; excitation energies of the lowest states corresponding to excitations from HOMO→LUMO (B_1u_), HOMO→LUMO+1 (A_u_), and HOMO–1→LUMO (B_2u_) are given in Table 1. For the sake of convenience, these states will be referred to as S_1_, S_2_, and S_3_, with the index referring to the energetic ordering of theoretical results. Within GAPW and relying on the B3LYP method, the CP2K results reproduce the TURBOMOLE reference data, validating the implementation of the interface. Switching from molecular basis sets to MOLOPT basis sets, optimized for PBC, introduces only small deviations of max. 0.07 eV. Relying, however, on the GGA functional PBE^84^ or switching to sTDA kernels reorders the excited states, with an excitation from HOMO–2→LUMO being lower in energy than the excitation to the B_2u_ state. The latter now corresponds to the fourth excited state with an excitation energy which is, by fortuitous error cancellation, closer to the experimental value, but differs from the B3LYP reference by 0.2–0.4 eV.

**Figure 2:**
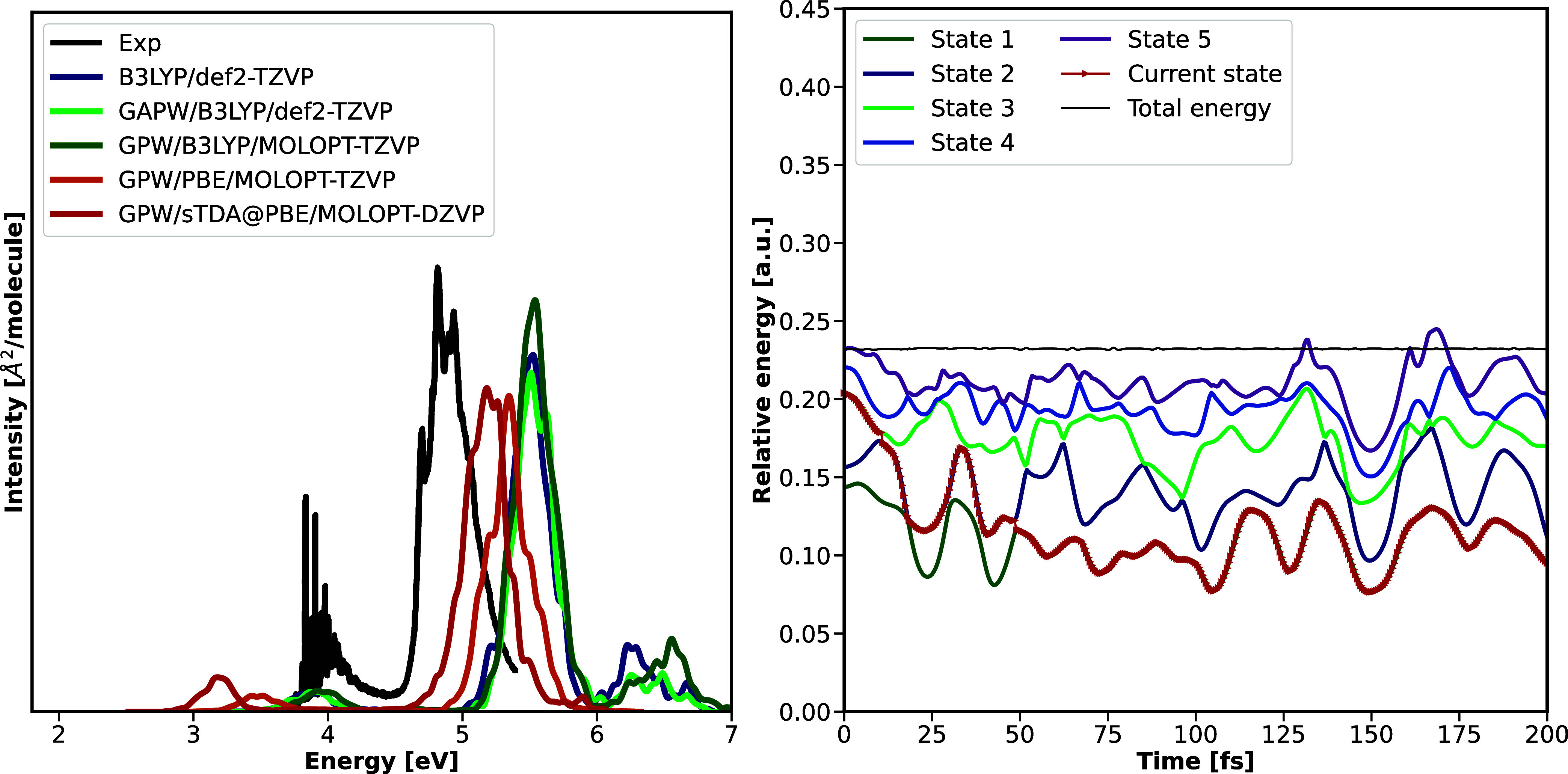
Left: Electronic absorption spectrum [in eV] of the lowest 5 excited states of pyrazine comparing implementations within the program packages TURBOMOLE and CP2K, using the B3LYP or PBE functional and an all-electron, molecular def2-TZVP or a MOLOPT-TZVP basis set, the latter in combination with GTH pseudo potentials. Experimental reference data is taken from ref 70 Right: Exemplary trajectory for molecular pyrazine based on OD couplings, the B3LYP functional and a molecular def2-TZVP basis set. Energies are reported in atomic units (a.u.).

**Table 1:** Excitation Energies [in eV] for the First Three Excited States of ^1^B_1u_ (HOMO→LUMO), ^1^A_u_ (HOMO→LUMO+1), and ^1^B_2u_ (HOMO–1→LUMO) of Molecular Pyrazine at Various Levels of Theory

setup		B_1u_, S_1_	A_u_, S_2_	B_2u_, S_3_ or S_4_
Exp.^64^		3.8	–	5.69
TURBOMOLE	B3LYP/TZVP	4.00	4.62	S_3_: 5.61
CP2K	GAPW/B3LYP/TZVP	4.00	4.62	S_3_: 5.59
	GPW/B3LYP/MOL-TZ	4.07	4.76	S_3_: 5.60
	GPW/PBE/MOL-TZ	3.60	4.05	S_4_: 5.42
	sTDA/PBE/MOL-DZ	3.27	3.95	S_4_: 5.32

Excited-state relaxation is therefore started from state S_4_ for PBE and sTDA@PBE kernels. Furthermore, the first B_1u_ and the dark A_u_ state are shifted to lower excitation energies of 3.60/3.27 and 4.05/3.95 eV for PBE and sTDA@PBE kernels, respectively. Further setups were tested, and corresponding results are given in the Supporting Information (Figure S2). The fact that GGA functionals reverse the energetic ordering of excited states in comparison to hybrid functionals underlines the importance of accurate XC functionals to describe excited states.^85^ It might thus be beneficial to test optimally tuned range-separated hybrid functionals, which outperform hybrids and were shown to have an accuracy for energy gaps, excited-state characters, and NACs comparable to semiempirically parametrized CAM-B3LYP or EOM-CCSD.^86^

To simulate excited-state relaxation, we performed nonadiabatic dynamics propagation of at least 200 trajectories for 200 fs, comparing time-dependent excited-state populations and lifetimes. An exemplary trajectory is visualized in Figure 2, highlighting that, starting from the second bright state ^1^B_2u_, relaxation to the lower dark state ^1^A_u_ is observed already within the first 10 fs, subsequently followed by a transition to the energetically lowest bright ^1^B_1u_ state. The latter is well separated from the ground state, so that no relaxation to the ground state is observed. Corresponding adiabatic populations are visualized in Figure 3, comparing the various choices for coupling constants (OD, TDBA, and LD with OD overlap matrix) that can be combined flexibly with the various quantum-mechanical setups (based on GAPW and GPW in combination with conventional TDA kernels or the simplified Tamm-Dancoff approximation (sTDA)). Additional plots further validating, e.g., the agreement between TURBOMOLE and CP2K or adding further setups with varying coupling constant - quantum mechanical method combinations are given in the Supporting Information (Figure S3). Populations plotted in Figure 3a, comparing CP2K and TURBOMOLE results for Baeck-An couplings, again, validate the interface with respect to the existing setups.

**Figure 3:**
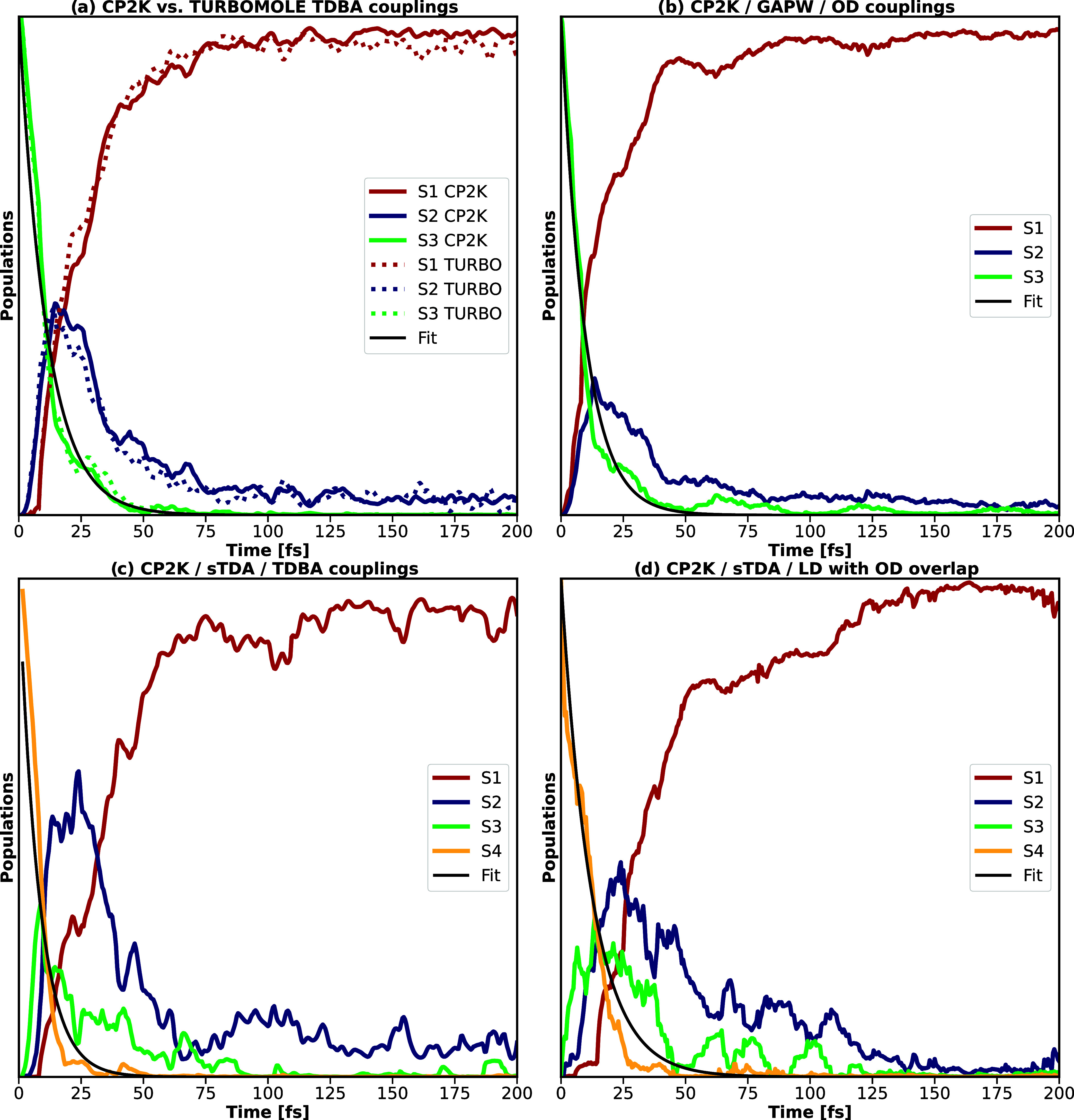
Time-dependent excited-state adiabatic populations as obtained by averaging over 200 nonadiabatic trajectories for pyrazine, validating the CP2K implementation with respect to TURBOMOLE, and comparing 1. the different coupling setups — (a) and (c) TDBA, (b) OD, (d) LD with OD overlap — as well as 2. the different quantum-mechanical kernels — (a) and (b) conventional GAPW, (c) and (d) simplified Tamm-Dancoff approximation (sTDA). All computations were performed using the B3LYP functional and a def2-TZVP basis set.

Furthermore, comparing the different coupling schemes, state populations agree well. However, even though the fitted lifetime constants of approximately 10 fs agree well among the different setups, they are too small in comparison to the literature values of 20–22 fs.^64,87–89^ Comparing the computational setup of the TURBOMOLE computations with ref 64 suggests that the main source of error is to be seen in the underlying coupling scheme, with DD couplings thus outperforming TDBA or OD couplings. Switching from conventional TDA to sTDA kernels (see Figure 3c,d) includes, due to starting from S_4_, subsequent population of S_3_ and S_2_, with similar population profiles for both OD and TDBA couplings, as well as local diabatization. Relaxation is, however, even more overestimated with lifetimes of approximately 5-12 fs. For all density-functional theory and semiempirical setups, relaxation to the ground state is not observed within the first 200 fs.^90^

Coupling magnitudes have been compared for OD couplings for CP2K and TURBOMOLE implementations, plotting the absolute coupling magnitude along a trajectory of 100 fs for the coupling of the two lowest excited states S_1_ and S_2_ as well as for the subsequent higher-lying excited states S_2_ and S_3_ for two different trajectories. The match in coupling magnitudes again validates the agreement of both implementations. A comparison of coupling magnitudes between different coupling setups, e.g., for OD vs TDBA couplings, is moreover given in Figure 4, providing an overall qualitative agreement, with the time and width of the couplings being well-reproduced. Absolute magnitudes are, however, underestimated by TDBA. The latter could be due to the chosen default setup for the TDBA parameters, with TDBA couplings being set to zero if the absolute energy gap of the involved states, *δε* = |Δ*E*_*MN*_(*t*)|, was larger than 2 eV. Benchmark results of ref 30 suggest that an improvement in coupling intensities could be achieved by optimizing those parameters. It thus has to be stressed that Figure 4 can not be referred to when aiming for a general discussion on the reliability of TDBA for molecular systems. Restricting the viewpoint to setting a nonoptimized reference for the comparison with results for crystalline pyrazine, implying periodic boundary conditions, this nonoptimized choice of parameters for TDBA couplings is, however, sufficient. Further benchmarking to determine an optimal parameter choice is not the focus of the current contribution and is therefore not discussed further.

**Figure 4:**
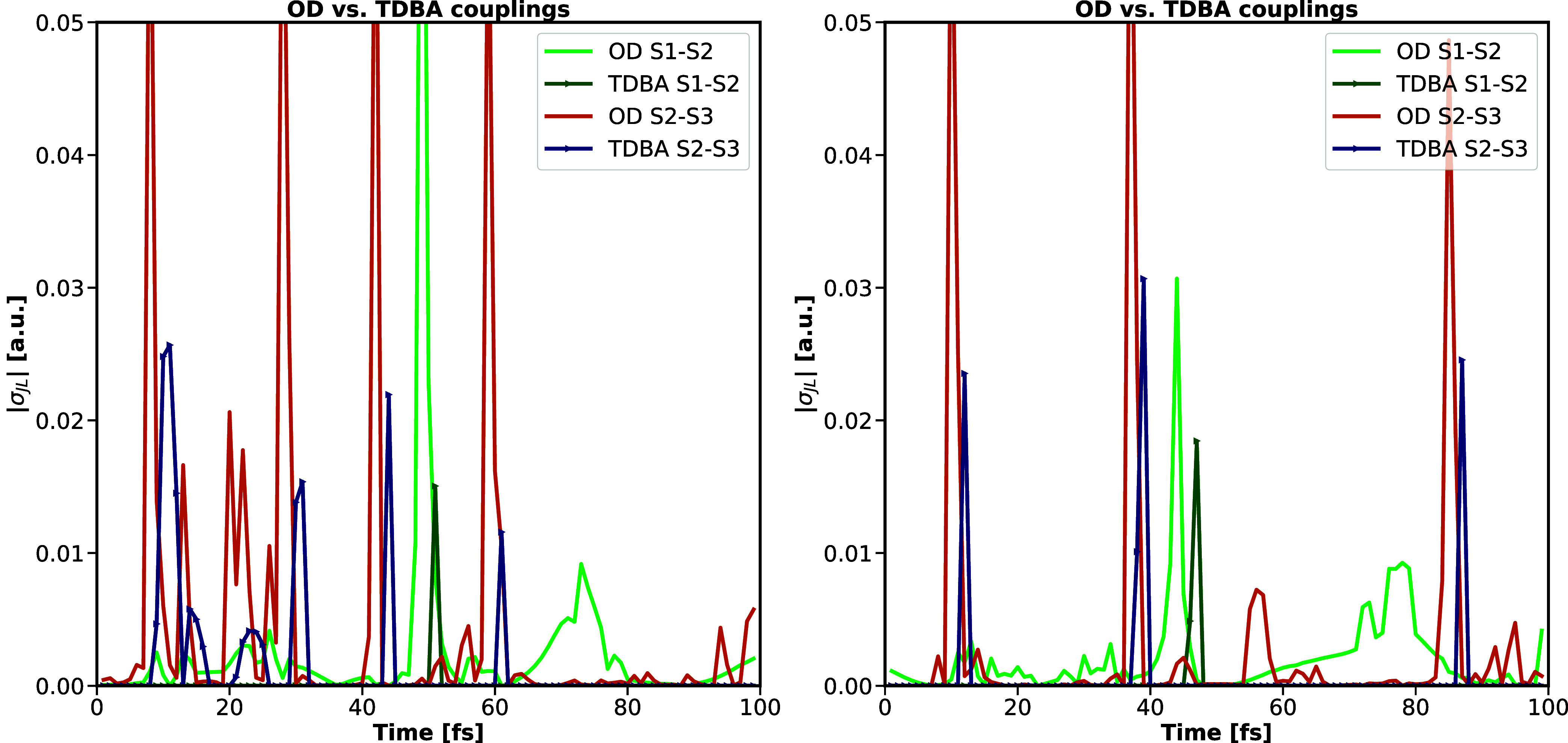
Comparison of coupling magnitudes |σ_*IJ*_| [in a.u.] for OD and TDBA couplings along two examplary trajectories of 100 fs for molecular pyrazine, visualizing couplings between the lowest B_1u_ and the dark A_*u*_ state (OD in orange, TDBA in blue) as well as the couplings between the second B_2u_ and the dark A_*u*_ state (OD in light green, TDBA in dark green).

### 3.2 Extension to Crystalline Pyrazine

Pure crystalline pyrazine does not show any fluorescence.^91^ Experimental spectroscopic data on crystalline pyrazine are restricted to phosphorescence,^91,92^ competing with quenching via triplet–triplet annihilation and showing lifetimes of the lowest triplet state in the range of 10^–2^ s. However, pyrazine-based dyes with modified functional groups are broadly used to tune fluorescence in the solid state,^93–96^ with aggregation-induced emission representing, e.g., a versatile tool to modify photophysical properties.^97^ Solid-state absorption and fluorescence properties were hereby found to be more sensitive to (electron-donating) abilities of functional groups due to crystallization. An extensive study on tuning triplet–triplet annihilation or exploiting aggregation-induced emission for different materials classes, such as functionalized pyrazine dyes, is beyond the scope of this contribution. For the current study, we limit the discussion to the broadened absorption spectrum of crystalline pyrazine in comparison to experimental reference data^98^ and demonstrate the usability and efficiency regarding computational timings of the outlined tool set.

Computational setups for the crystal are now consistently relying on the Gaussian and plane wave (GPW) method, using MOLOPT basis sets^80^ and Goedecker-Teter-Hutter pseudopotentials.^81,99^ 80 excited states were considered for the simulations in total. While different basis set sizes and functionals were tested for the computation of the broadened absorption spectrum, the setup was restricted, for the sake of convenience, for the surface hopping computations to sTDA kernels and relying on PBE^84^ references generated with MOLOPT-DZVP basis sets. In this case, 91 surface hopping trajectories of 100 fs were computed. While this sample size is smaller than the 200 trajectories run for molecular pyrazine, simulations were checked to be converged with respect to the number of trajectories. To avoid artifacts due to the high density of states,^100^ the nuclear time step was reduced to 0.1 fs in comparison to molecular pyrazine. All pairwise couplings between all 80 excited states were computed at each time step rather than restricting to adjacent-state interactions. The crystal structure is visualized in the Supporting Information (Figure S1) with the unit cell comprising 40 atoms.

Figure 5 visualizes on the left-hand side the broadened absorption spectrum for the various computational setups as well as experimental measurements. Plotted experimental data were taken from ref 98 and discriminate between the absorption spectrum obtained with light incident perpendicular to the (101) crystal face (denoted as Exp ⊥), spectra obtained from projection onto the (100) crystal face (denoted as Exp ∥) as well as the solution spectrum in hexane. The experimental spectrum thereby exhibits two distinct broad peaks: a smaller one from 3.7 to 4.2 eV and a larger one from 4.2 to 5.2 eV, peaking at 4.7 eV. The computational results for the 80 lowest states cover an energy range of 2 to 7 eV, with the relatively broad absorption peak being centered for the PBE functional between 4.7 to 4.9 eV, only slightly depending on the chosen basis set. The center of the analogous B3LYP band is shifted to 5.5 eV and moreover shows a shoulder at 4.9 eV. Switching from conventional to sTDA kernels has a negligible effect on the overall spectrum with similar band widths and heights.

**Figure 5:**
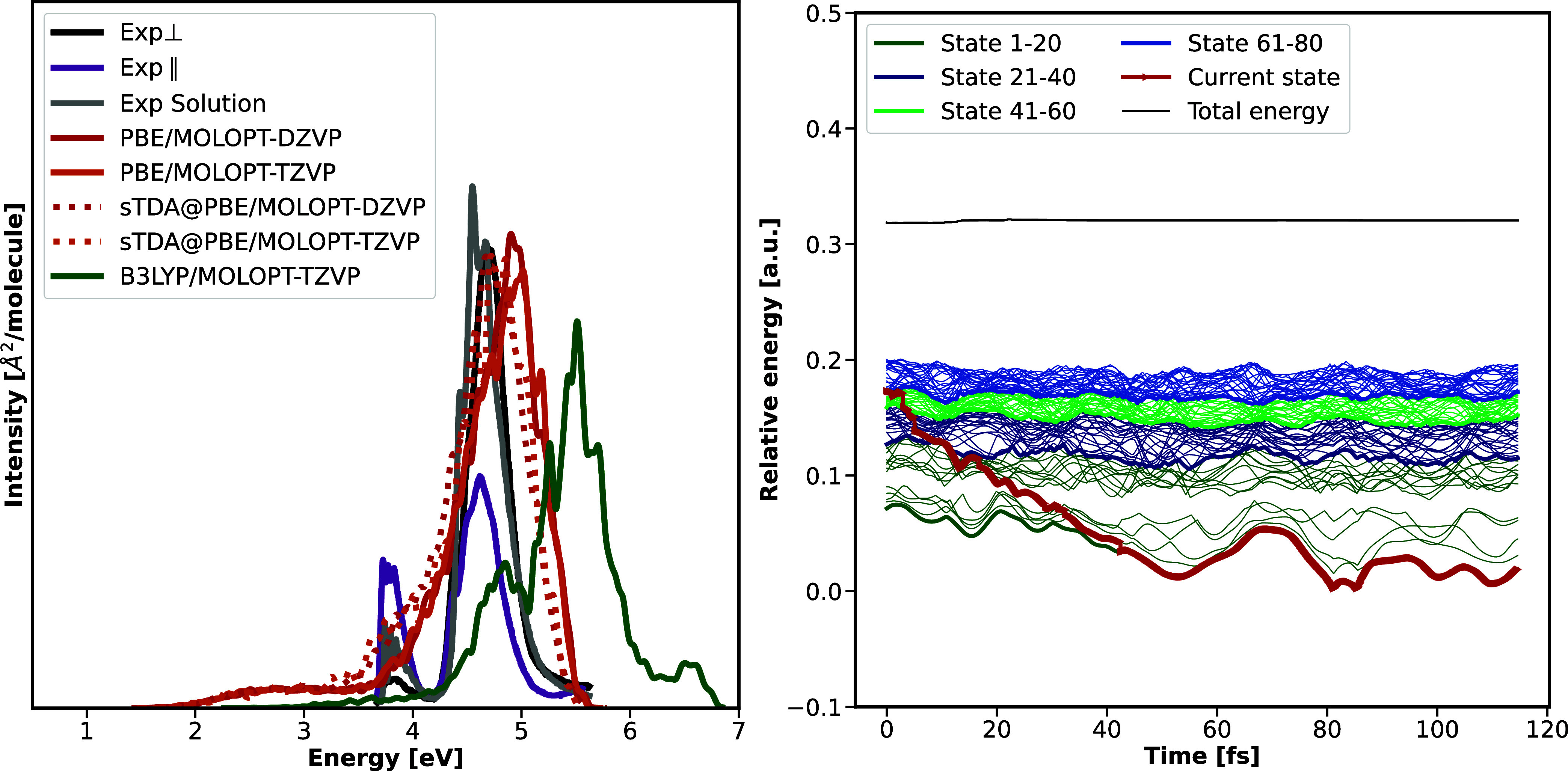
Left: Electronic absorption spectrum of the lowest 80 excited states of crystalline pyrazine, using the B3LYP or PBE functional or sTDA@PBE kernels, a MOLOPT-TZVP or MOLOPT-DZVP basis set and GTH pseudo potentials. Experimental reference data^98^ discriminates between the absorption spectrum obtained with light incident perpendicular to the (101) crystal face (Exp ⊥), spectra obtained from projection onto the (100) crystal face (Exp ∥) as well as the solution spectrum in hexane (Exp Solution). Right: Exemplary trajectory for crystalline pyrazine based on orbital derivative couplings, an sTDA kernel, the PBE functional, and a MOLOPT-DZVP basis set. Excited states of higher energy are so dense that single trajectories cannot be distinguished. To nevertheless associate bands with a range of excited states, color coding is adjusted, always highlighting 20 excited states with the same color.

When comparing energies of the highest 10 excited states (71st to 80th), the energy differences nevertheless amount to 0.5 eV. Since the first benchmark results for molecular pyrazine indicated that semiempirical GFN1-xTB ground-state references need to be tuned, i.e., by shifting the virtual orbital space (see Figure S2 of the Supporting Information), an extension of the benchmark to further modify the underlying ground-state reference is beyond the scope of the current investigation. It should, however, be stressed that optimized GFN1-xTB references could further increase the computational efficiency by an order of magnitude and that the suitability of such setups was already tested and approved.^35,36,43^ The band peak for sTDA kernel and PBE references corresponds to the 60th or 61st excited state, with both states being thus used to generate starting velocities and geometries, and the manifold of trajectories to obtain averaged adiabatic populations thus relies on the inputs obtained from these two states.

An exemplary trajectory for OD couplings is visualized in Figure 5 on the right-hand side, highlighting the increased density of states as well as the fast relaxation, consecutively over the intermediate excited states, to the first excited state S_1_ within the first 40 fs. Similar plots are obtained for LD couplings.

Resulting adiabatic populations, obtained by averaging over 91 trajectories, confirm this finding (Figure 6): All trajectories exhibit ultrafast internal conversion toward the first excited state S_1_ with most population being transferred within 40–50 fs. For OD couplings, the high-lying states are quickly depopulated and, in return, occupation of increasingly lower excited states sets in, with a maximum population of the 20th excited state being found after ∼8 fs and of the 10th excited state after ∼25 fs, respectively. The population of the three lowest excited states increases after 20 fs, with the third and second excited states reaching a maximum at 40 fs and then slowly decreasing to below 5%. While the first excited state reaches a maximum population of 95%, no relaxation to the ground state is observed within the total simulation time of 100 fs. LD based on the OD overlap matrix yields a similar picture: After 100 fs, the first excited state reaches an average population of 90%, with the relaxation proceeding over lower lying states reflected in the subsequent maxima in respective populations.

**Figure 6:**
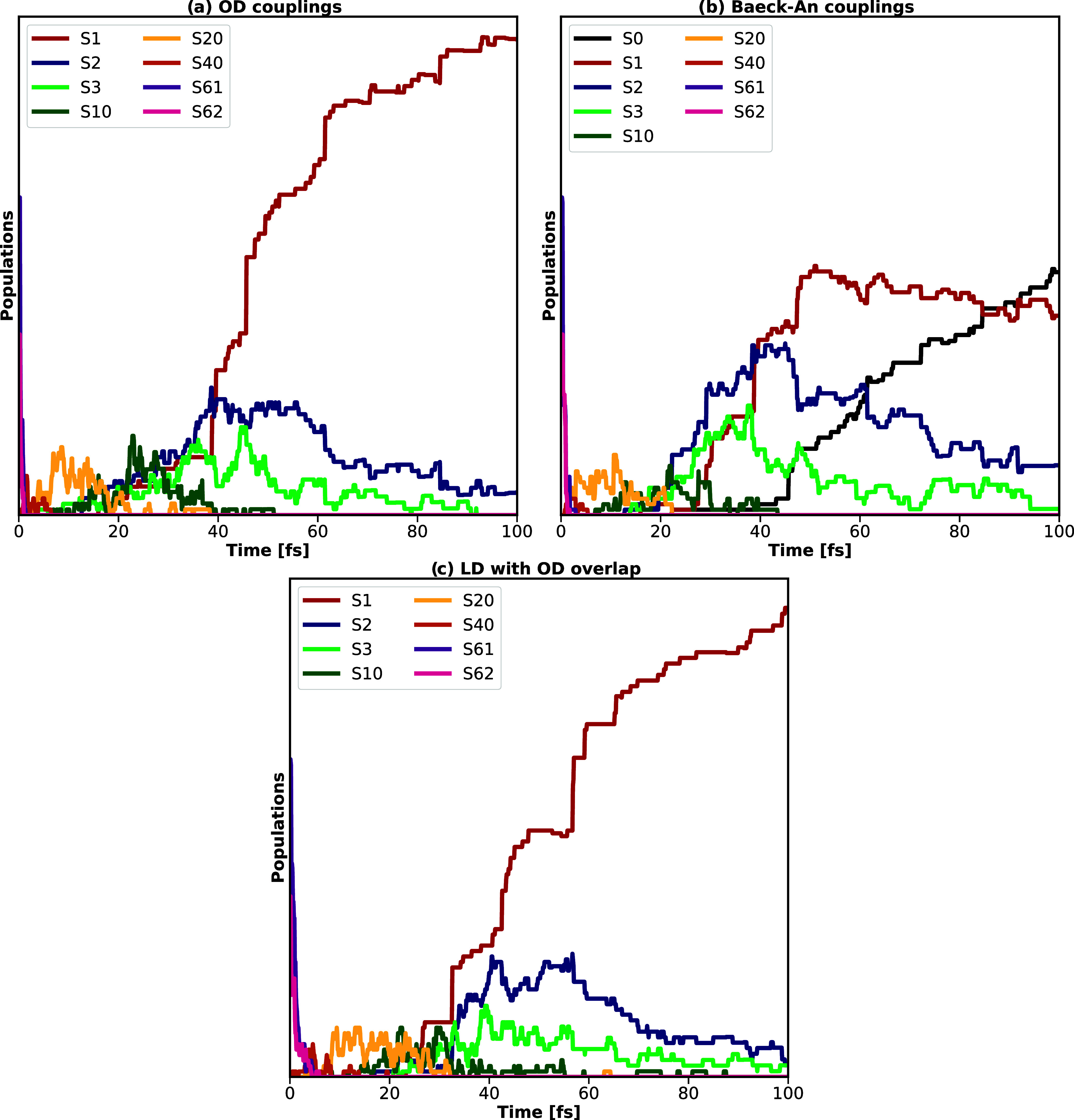
Time-dependent adiabatic populations as obtained by averaging over 91 nonadiabatic trajectories for crystalline pyrazine, relying on (a) OD, (b) phenomenological time-dependent Baeck-An couplings or (c) local diabatization, sTDA, the PBE functional and a MOLOPT-DZVP basis set. TDBA leads to the artifact that the ground state is populated, with the corresponding population thus being additionally given in subfigure (b).

Regarding analogous simulations relying on time-dependent Baeck-An couplings, it needs to be stressed that, as discussed within ref 30, the model is formally only valid to describe the coupling of two states. Computations were thus performed based on the assumption that two-state coupling is adequate also for crystalline pyrazine. Due to the much higher density of states, the TDBA input parameter *δε* had to be adjusted accordingly to yield similar relaxation profiles within the first femtoseconds. Best agreement was found when setting TDBA couplings to zero if the absolute energy gap of the involved states, *δε*, was larger than 0.5 eV. As documented in Figure S5 of the Supporting Information, other variations of *δε* result in increasing or decreasing relaxation times; further modification of input parameters, to, e.g., guarantee smoothness in the variation of the couplings, did not improve the results. As visualized in graph b of Figure 6, relaxation is then comparable to OD couplings for the first 40 fs. After 46 fs, however, the electronic-state population of the ground-state increases above 0.1%, with steadily decreasing population of the three lowest excited states.

While the increasing population of the ground state flaws the performance of TDBA couplings, a comparison of coupling magnitudes for OD and TDBA couplings, as given in Figure 7, nevertheless reveals the overall quantitative agreement of both ansätze when restricting the assessment to internal conversion within the manifold of excited states (see also additional data of Figure S4 in the Supporting Information). Couplings between excited states S_1_ and S_2_ are consistently smaller than 0.02 a.u for both coupling models. In contrast, the interaction between excited states S_2_ and S_3_ is for both OD and TDBA systematically larger, even though TDBA underestimates corresponding OD coupling magnitudes.

**Figure 7:**
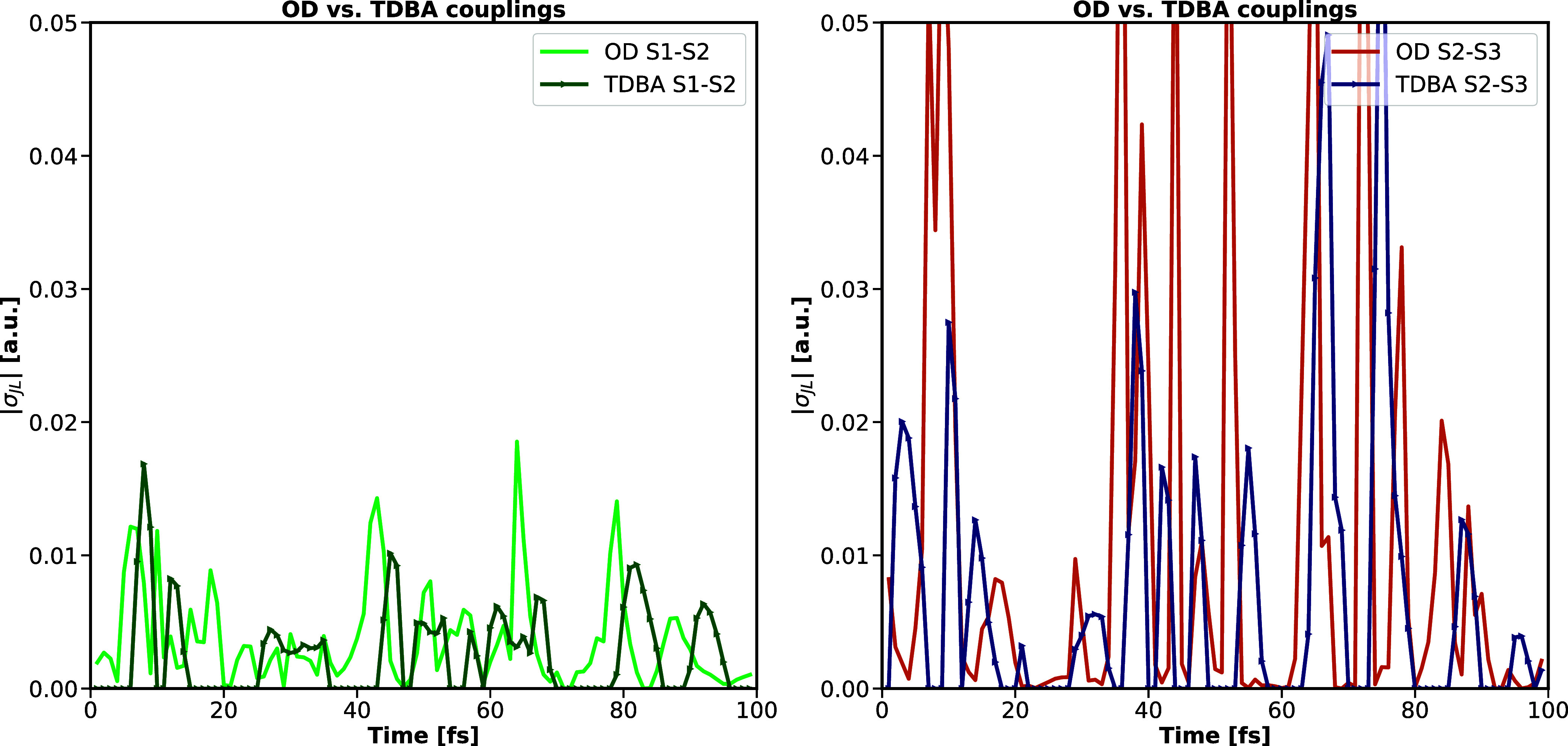
Comparison of coupling magnitudes |σ_*IJ*_| [in a.u.] for OD and TDBA couplings along a typical trajectory of 100 fs for crystalline pyrazine, visualizing couplings between excited states S_1_ and S_2_ (left-hand side; OD in light green, TDBA in dark green) as well as the couplings between excited states S_2_ and S_3_ (right-hand side; OD in orange, TDBA in blue).

In total, one can thus conclude that OD and LD yield similar population patterns, with S_1_ reaching a population of ≈90–95%. TDBA couplings, in contrast, exhibit spurious decay to the ground state, even though a comparison of coupling magnitudes for relaxation within the excited-state manifold provides qualitative agreement with OD couplings.

The CP2K-NEWTON-X interface is set up with the motivation to provide sufficient computational efficiency for large-scale computations on extended systems. Based on the outlined preliminary assessment of different setups for molecular and crystalline pyrazine, computational timings representing the thereon based main result of this manuscript are summarized in Table 2 for crystalline pyrazine, referring again to a unit cell comprising 40 atoms corresponding to 416 basis functions for a MOLOPT-DZ basis. Computations were performed on an AMD EPYC GENOA 9534 processor utilizing a total of 64 cores. For the electronic structure computation within CP2K computing 80 excitation energies as well as the gradient for the 61st excited state, the total computation time amounts to 30 s for MOLOPT-DZ basis sets, PBE references, and sTDA kernels, assuming tight convergence criteria of 10^–9^ a.u., as well as grid cutoffs of 800 Ry. Computing corresponding nonadiabatic coupling constants within the orbital derivative ansatz adds another minute to the total computation time of 1 min 57 s, demonstrating that the method is tractable even for crystalline systems. Relying on TDBA couplings or local diabatization yields comparable computation times of 1 min 43 s and 1 min 35 s, respectively. Computational costs increase by a factor of 5 when switching to conventional TDA kernels using GGA functionals and are reduced by a factor of 2 for semiempirical GFN1-xTB references.

**Table 2:** Timings for Performing a NAMD Step for Crystalline Pyrazine Considering the Lowest 80 Excited States^a^

	TDA	sTDA	sTDA
timing	PBE/MOL-DZ	PBE/MOL-DZ	GFN1-xTB
CP2K	3 min 55 s	30 s	10 s
OD overlap	1 min 2 s	1 min 2 s	11 s
total OD	5 min 25 s	1 min 57 s	45 s
total TDBA	5 min 4 s	1 min 43 s	38 s
total LD	5 min 35 s	1 min 35 s	23 s

a Computations were performed on an AMD EPYC GENOA 9534 processor using 64 cores.

Further computational timings for the underlying electronic structure computation were already reported in ref 57 It highlights that sTDA in general enables an order of magnitude in speedup compared to ADMM-approximated hybrid functional kernels. Thus, it is most beneficial when aiming to compute broadband spectra, which implies a large number of excited states. Relying on sTDA kernels based on GGA functionals, even larger unit cell sizes of hundreds of atoms should thus be accessible at a moderate computational cost, with timings in the order of minutes to compute both broad-band electronic excitation spectra as well as the corresponding excited-state nuclear gradient.

## 4 Conclusions

With the outlined variety of time-dependent density functional theory and semiempirical approximations for electronic-structure methods and the range of time-dependent Baeck-An to numerical couplings for the trajectory surface hopping, the current contribution establishes the interface of the electronic-structure program package CP2K^51,53^ and the surface-hopping code NEWTON-X^48–50^ as a versatile tool set for nonadiabatic molecular dynamics on extended materials, implying periodic boundary conditions.

Assessed features from the electronic-structure program package comprise conventional TDDFT based on GGA as well as hybrid functionals, the latter accelerated with ADMM. Semiempirical sTDA complements the electronic-structure setup, which can be used relying on conventional DFT references or on semiempirical GFN1-xTB references, with the latter, however, requiring further adjustments and benchmarking. Both excited-state kernels can be combined either with GPW or GAPW. NEWTON-X modularly provides phenomenological time-dependent Baeck-An, numerical orbital time-derivative couplings, or enables the use of local diabatization based on the orbital derivative overlap matrix.

Validation of the interface was demonstrated for molecular pyrazine, highlighting the close comparison to the molecular electronic structure code TURBOMOLE^59,60^ and demonstrating the adequacy of the mentioned approximations for coupling constants to describe excited-state relaxation from the second to the first excited state. The obtained results reflect the error range of the density functional theory setup, with excitation bands being off by 0.2 to 0.8 eV for the investigated lowest excited states, respectively. Lifetimes of 5–12 fs underestimate the experimental reference value of 22 fs.

Analogous computations for crystalline pyrazine underscore the agreement between lifetimes obtained for OD couplings and corresponding results relying on local diabatization, demonstrating the efficiency of the interface. This enables the performance of a NAMD step with a semiempirical TDDFT kernel, a DFT ground-state reference, and a double-ζ basis set within minutes. While both local diabatization as well as OD and TDBA couplings yield similar results for molecular pyrazine, care must be taken when using TDBA couplings for crystalline pyrazine, implying periodic boundary conditions and a comparably high density of states. In this case, the two-state nature of the TDBA model leads to population leakage into the ground state. Future work will be focused on extending the tool set to enable a more extensive and accurate description of excited-state phenomena, including e.g., intersystem crossing.

## Supplementary Material





## Notes

Structural information on pyrazine crystal; visualization of structural data broadened absorption spectra for molecular pyrazine; adiabatic populations for molecular pyrazine; coupling magnitudes for crystalline pyrazine; adiabatic populations for TDBA couplings for crystalline pyrazine (PDF)
Additional information (ZIP)
