# Supplementary material for: A Density Functional Theory and Semiempirical Framework for Trajectory Surface Hopping on Extended Systems
Source: J Chem Theory Comput. 2025 Oct 17;21(20):10474–88. doi: 10.1021/acs.jctc.5c01082 (PMC12573750; doi:10.1021/acs.jctc.5c01082)
Supplement: Supplementary file 1 [file ct5c01082_si_001.pdf]

Supplementary information: A density functional theory and semi-empirical framework for trajectory surface hopping on extended systems

Jan-Robert Vogt,[†] Michael Schulz,[†] Rafael Souza Mattos,[‡] Mario Barbatti,[‡,¶] Maurizio Persico,[§] Giovanni Granucci,[§] Jürg Hutter,[∥] and Anna Hehn[*,†,⊥]

†*Christian-Albrechts-University Kiel, Max-Eyth-Strasse 1, 24118 Kiel, Germany*

‡*Aix Marseille University, CNRS, ICR, 13397 Marseille, France*

¶*Institut Universitaire de France, 75231 Paris, France*

§*Dipartimento di Chimica e Chimica Industriale, Via Moruzzi 13, 56124 Pisa, Italy*

∥*Department of Chemistry, University of Zurich, Winterthurerstrasse 190, 8057 Zurich, Switzerland*

⊥*Kiel Nano, Surface and Interface Science, 24118 Kiel, Germany*

E-mail: hehn@pctc.uni-kiel.de

TABLE OF CONTENTS

**1.**	**Structural information on pyrazine crystal**	S3
**2.**	**Visualization of structural data**	S4
**3.**	**Broadened absorption spectra for molecular pyrazine**	S5
**4.**	**Adiabatic populations for molecular pyrazine**	S6
**5.**	**Coupling magnitudes for crystalline pyrazine**	S7
**6.**	**Adiabatic populations for TDBA couplings for crystalline pyrazine**	S8

To provide further information, Cartesian coordinates as well as a visualization of the pyrazine crystal structure are provided. Results for further computational setups to compute the broadened absorption spectrum of molecular pyrazine are reported as well as corresponding adiabatic populations for the thereon relying NAMD simulations. A comparison of coupling magnitudes for an examplary trajectory is provided for crystalline pyrazine. Furthermore, adiabatic populations for TDBA couplings are discussed in more detail for crystalline pyrazine investigating the influence of the threshold, which sets the couplings to zero depending on the absolute energy gap of involved states.

1 Structural information on pyrazine crystal

Table S1: Cartesian coordinates for crystalline pyrazine [in Å].

N	0.05857617	0.11519773	2.60387925
C	1.20073381	1.83941702	1.16842753
H	2.14600978	3.38523222	2.12494336
C	-1.25919948	-1.58390361	1.28086943
H	-2.14024478	-3.11139344	2.32687401
N	8.49416218	3.41621619	1.61366133
C	10.34449257	1.72382201	1.90689745
H	10.02487862	0.21275872	3.25607716
C	8.98661403	5.12887728	-0.16670366
H	7.64450657	6.66136166	-0.38898287
N	-5.08583861	3.74101847	6.48772200
C	-6.22903423	5.44227671	7.95412230
H	-7.24847197	6.95860937	7.02525086
C	-3.74216895	2.05133728	7.79654722
H	-2.83054584	0.55417640	6.73047275
N	-13.06409273	7.14552326	8.01565774
C	-14.95654058	5.53975109	7.57174085
H	-14.72968976	4.23734765	5.99339581
C	-13.51776848	8.76494271	9.88931799
H	-12.01699545	10.07454462	10.39385107
N	-0.27716354	0.17706010	-2.78107026
C	-1.58143297	-1.43285732	-1.35097514
H	-2.92156121	-2.68403718	-2.28513315
C	1.19372822	1.76248298	-1.48755699
H	2.40263670	3.02593795	-2.57588157
N	-8.20728579	-3.77584280	-1.29723671
C	-10.10956981	-2.17062498	-1.69907976
H	-9.91891235	-0.86502059	-3.27879090
C	-8.62011979	-5.39711548	0.58492338
H	-7.10683575	-6.70406428	1.05878020
N	4.84302117	-3.47286439	-6.72405544
C	6.09792651	-5.09602358	-8.18814243
H	7.37204068	-6.41958485	-7.26476351
C	3.39832750	-1.85808098	-8.01441252
H	2.26034549	-0.53999750	-6.91869916
N	13.12875511	-7.29657212	-7.78544661
C	14.96993198	-5.60096047	-7.45646734
H	14.62208740	-4.08943892	-6.11582627
C	13.65944856	-9.00653639	-9.55941405
H	12.32106819	-10.53715722	-9.81666551

2 Visualization of structural data

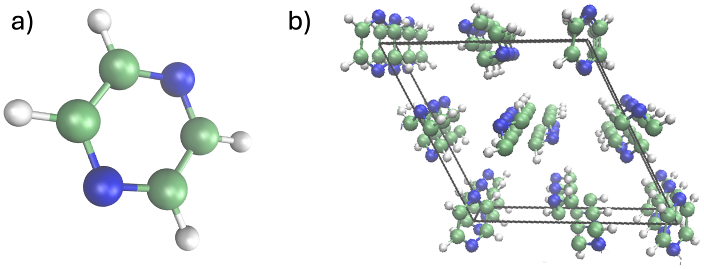

Figure S1: Visualization of a) molecular and b) crystalline pyrazine structures.

3 Broadened absorption spectra for molecular pyrazine

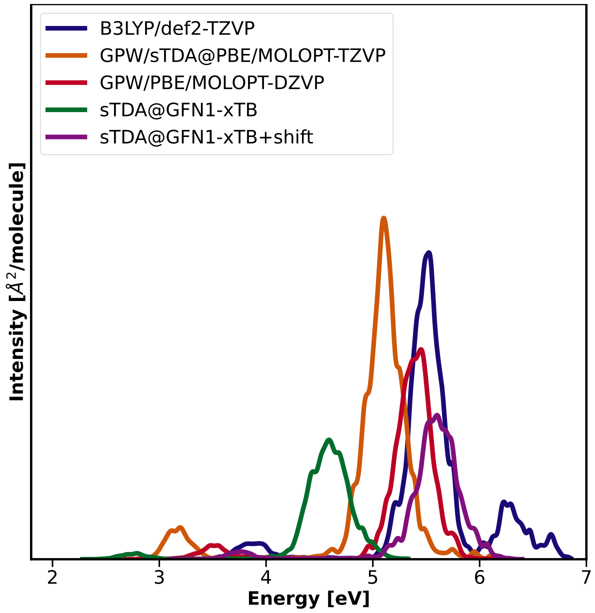

Figure S2: Electronic absorption spectrum of the lowest 5 excited states of pyrazine comparing implementations within the program packages Turbomole and CP2K, using the B3LYP or PBE functional and an all-electron, molecular def2-TZVP or a MOLOPT-TZVP basis set, the latter in combination with GTH pseudo potentials. The setups complement results visualized in Figure 2 of the main manuscript and suggest that semi-empirical references could be used if optimized e.g. by adding a shift of the virtual orbital space.

4 Adiabatic populations for molecular pyrazine

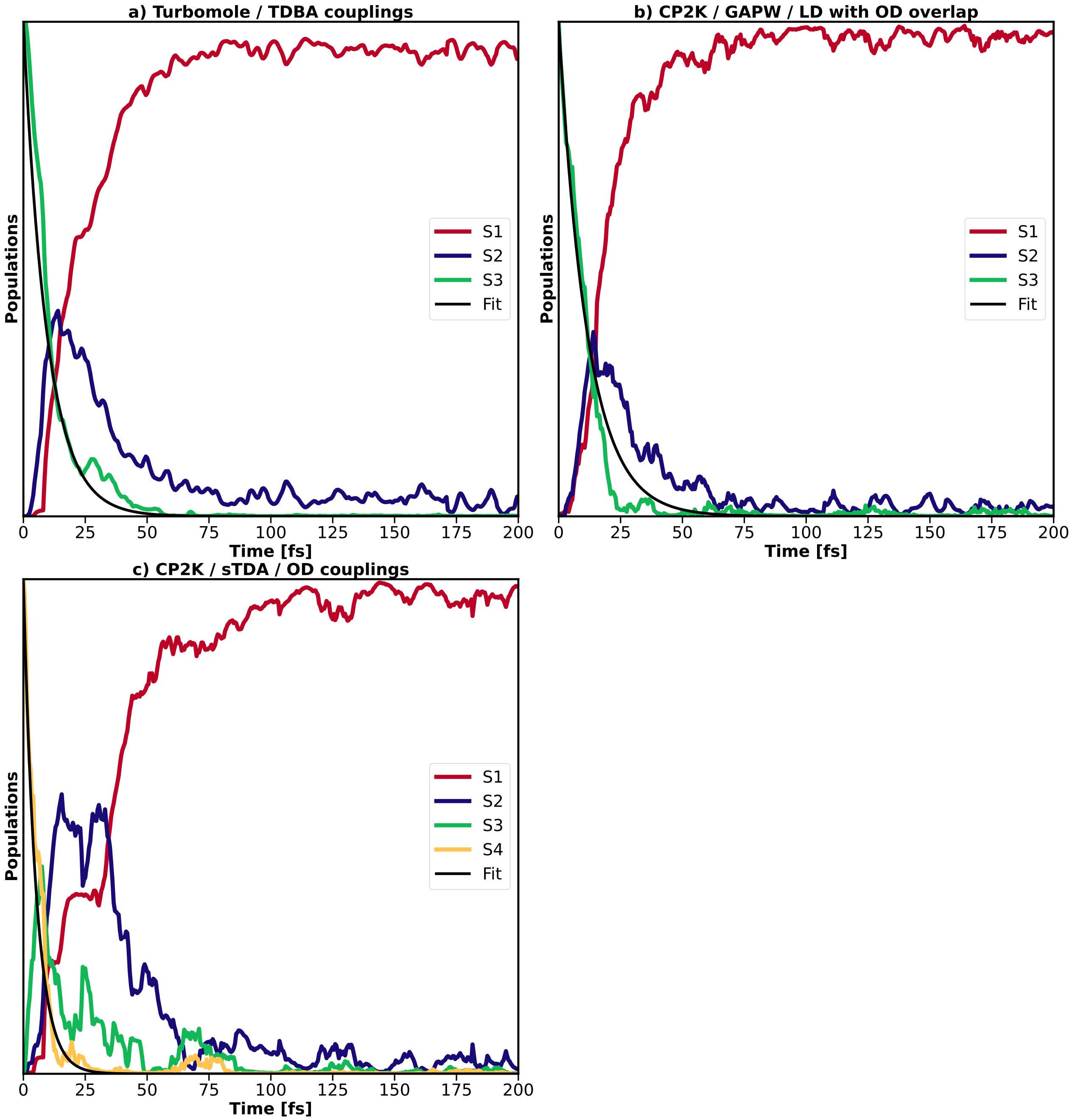

Figure S3: Time-dependent excited-state adiabatic populations as obtained by averaging over at least 200 non-adiabatic trajectories for pyrazine for further computational setups, based on the implementation in Turbomole (a), relying on a conventional (GAPW) kernel (b) or on simplified TDA (sTDA) kernels (c), as well as using OD (c), TDBA (a) couplings or LD based on OD overlap matrices (b). Either a B3LYP or a PBE reference was used combined with either a def2-TZVP or MOLOPT-DZVP basis.

5 Coupling magnitudes for crystalline pyrazine

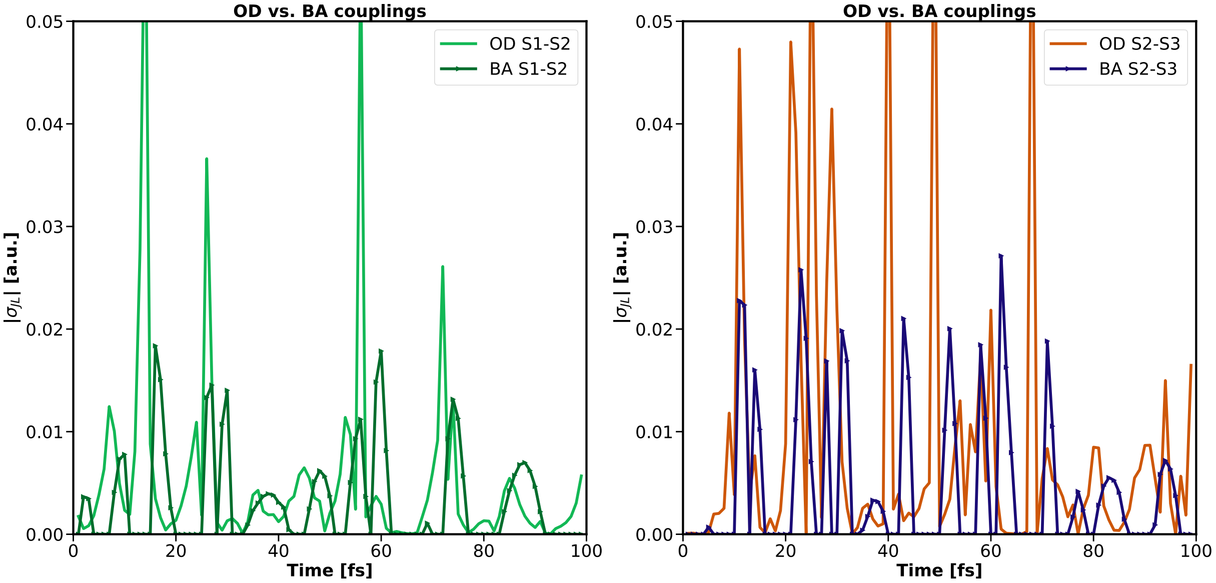

Figure S4: Comparison of coupling magnitudes $|\sigma_{IJ}|$ [in a.u.] for OD and BA couplings along a typical trajectories of 100 fs for crystalline pyrazine, visualizing couplings between state S1 and S2 (OD in light green, BA in dark green) as well as the couplings between state S2 and S3 (OD in orange, BA in blue).

6 Adiabatic populations for BA couplings for crystalline pyrazine

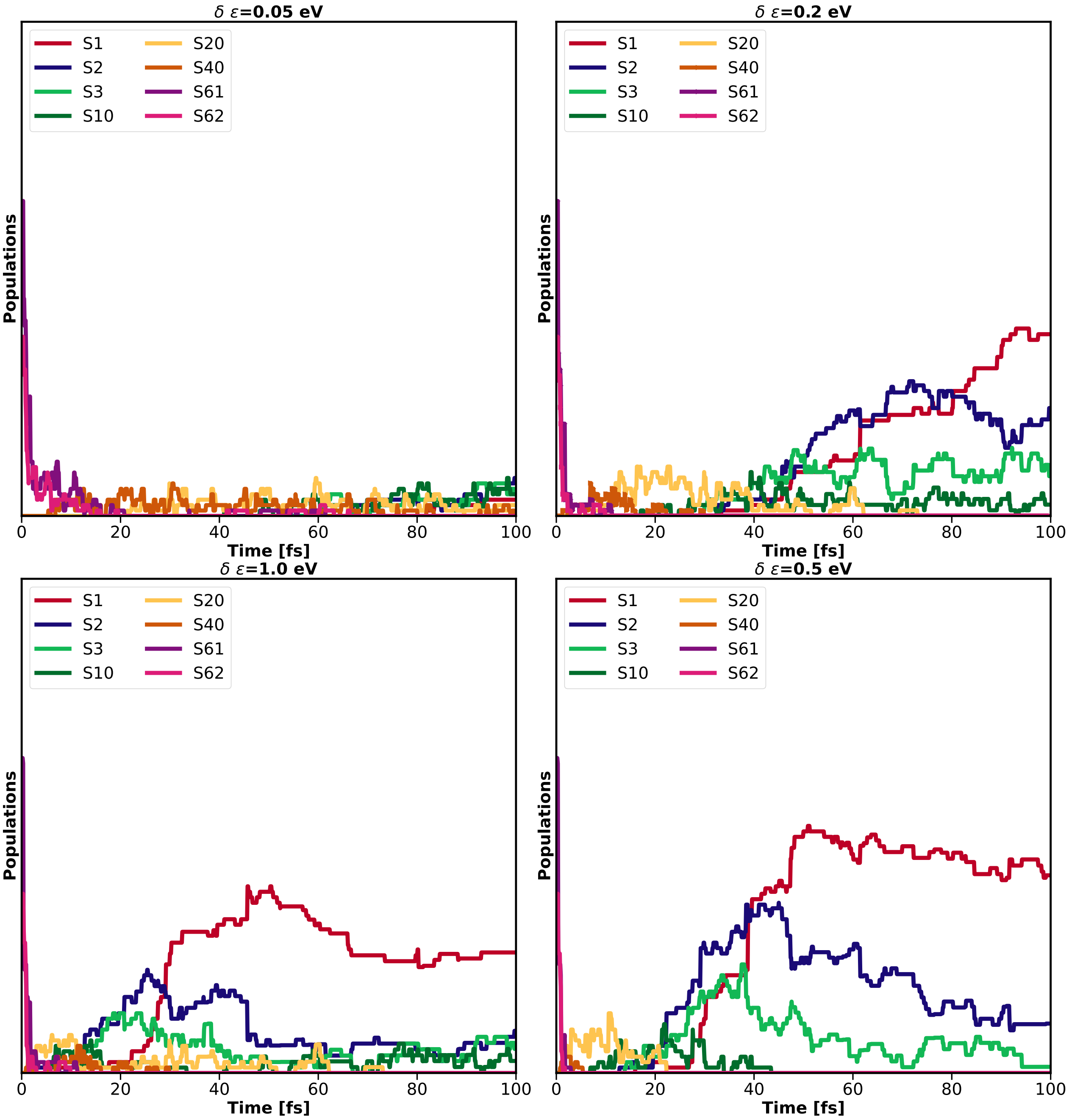

Figure S5: Time-dependent excited state adiabatic populations as obtained by averaging over at least 90 non-adiabatic trajectories for crystalline pyrazine comparing different Baeck-An parameterizations. Couplings are set to zero if larger than $\delta\varepsilon$ [in eV].
