# Supplementary material for: A Density Functional Theory and Semiempirical Framework for Trajectory Surface Hopping on Extended Systems
Source: J Chem Theory Comput. 2025 Oct 17;21(20):10474–88. doi: 10.1021/acs.jctc.5c01082 (PMC12573750; doi:10.1021/acs.jctc.5c01082)
Supplement: Supplementary file 2 [file ct5c01082_si_002.zip › Supplementary_information/PICTURES/bands_pyrazine_final_graph_zoom_in_NEW_with_xtbshift_for_SI.pdf]

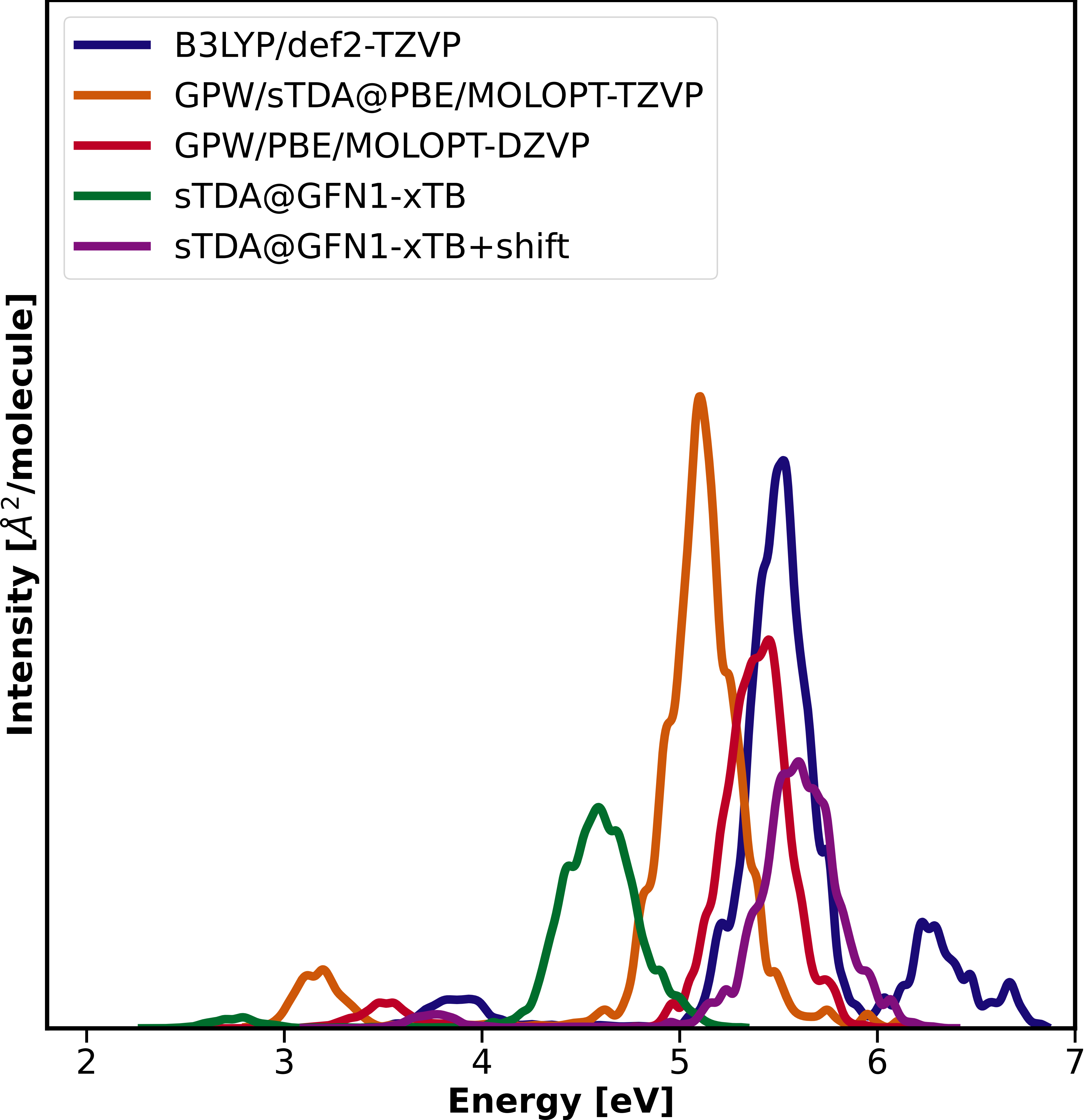
B3LYP/def2-TZVP
GPW/sTDA@PBE/MOLOPT-TZVP
GPW/PBE/MOLOPT-DZVP
sTDA@GFN1-xTB
sTDA@GFN1-xTB+shift
Intensity [Å²/molecule]
2
3
4
5
6
7
Energy [eV]
