# Supplementary figures and images for: A Density Functional Theory and Semiempirical Framework for Trajectory Surface Hopping on Extended Systems
Source: J Chem Theory Comput. 2025 Oct 17;21(20):10474–88. doi: 10.1021/acs.jctc.5c01082 (PMC12573750; doi:10.1021/acs.jctc.5c01082)

**a) Turbomole / TDDBA couplings**

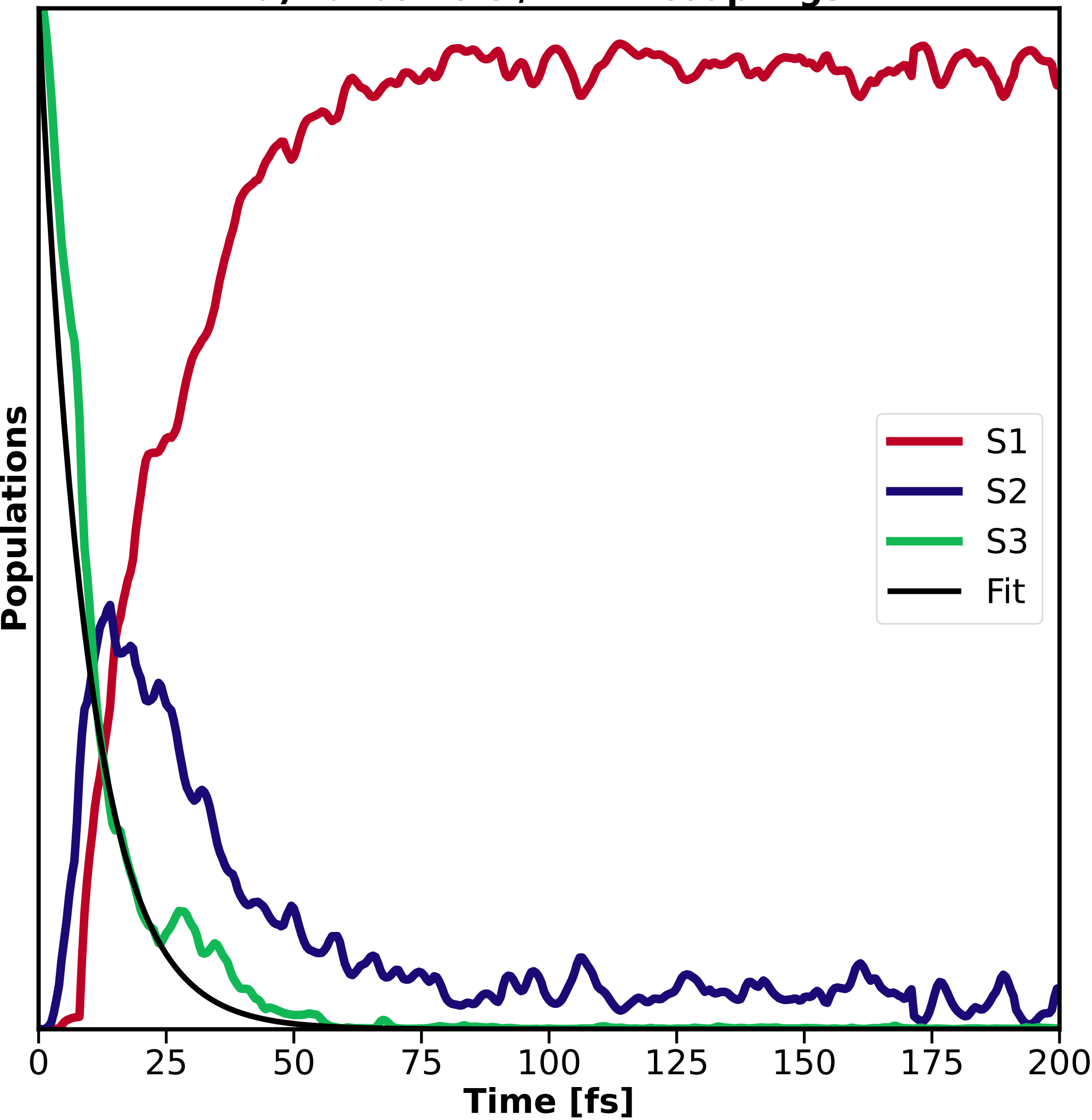

Supplement: Supplementary file 2 [file ct5c01082_si_002.zip › Supplementary_information/PICTURES/population_pyrazine_turbo_b3lyp_def2tzvp_with_adiabatic_population_fit_2.pdf]

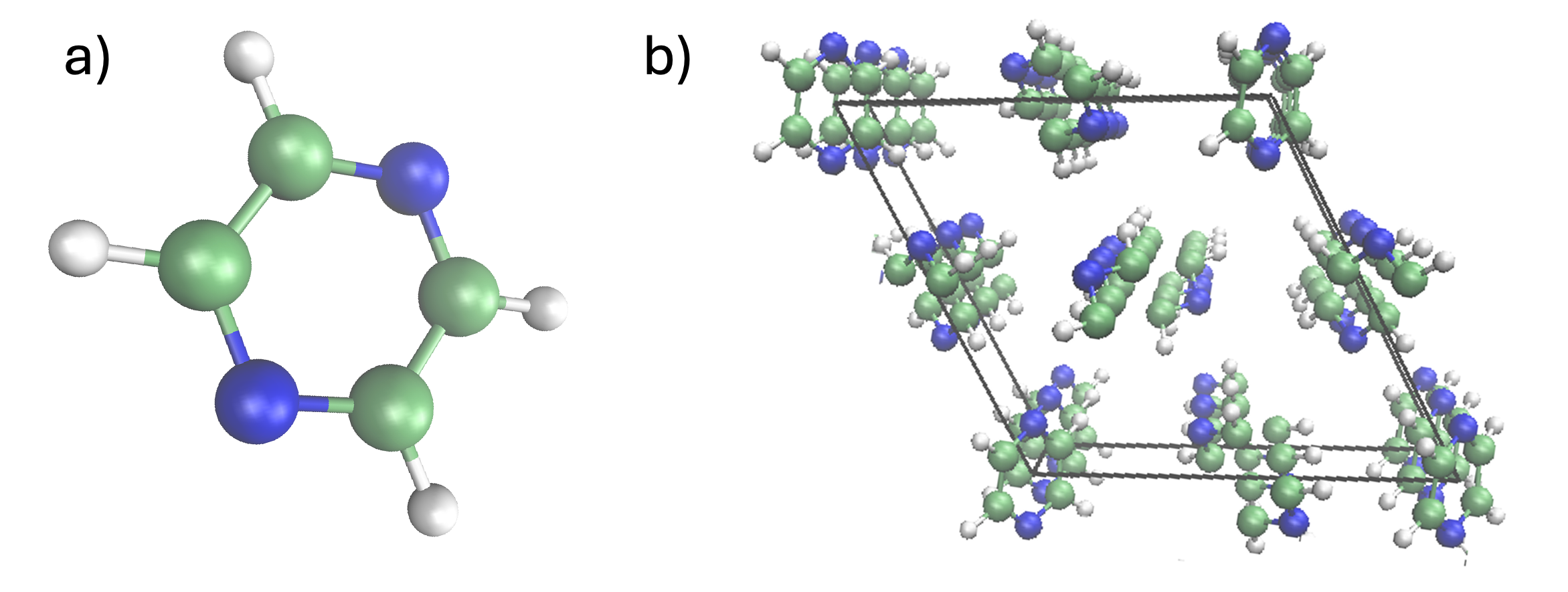

Supplement: Supplementary file 2 [file ct5c01082_si_002.zip › Supplementary_information/PICTURES/Pyrazine_pic.png]

$\delta \varepsilon = 0.5 \text{ eV}$

Populations

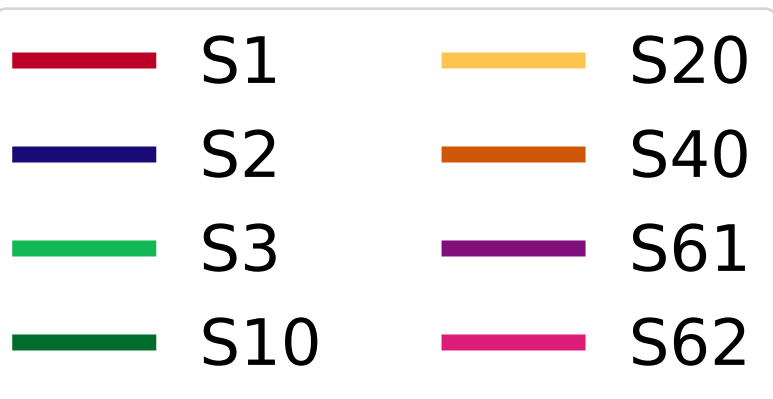

0 20 40 60 80 100

Time [fs]

Supplement: Supplementary file 2 [file ct5c01082_si_002.zip › Supplementary_information/PICTURES/population_pyrazine_pbe_crystal_molopt_dzvp_with_adiabatic_population_BA_modified_NEW_coupling_strength_of_05.pdf]

**OD vs. BA couplings**

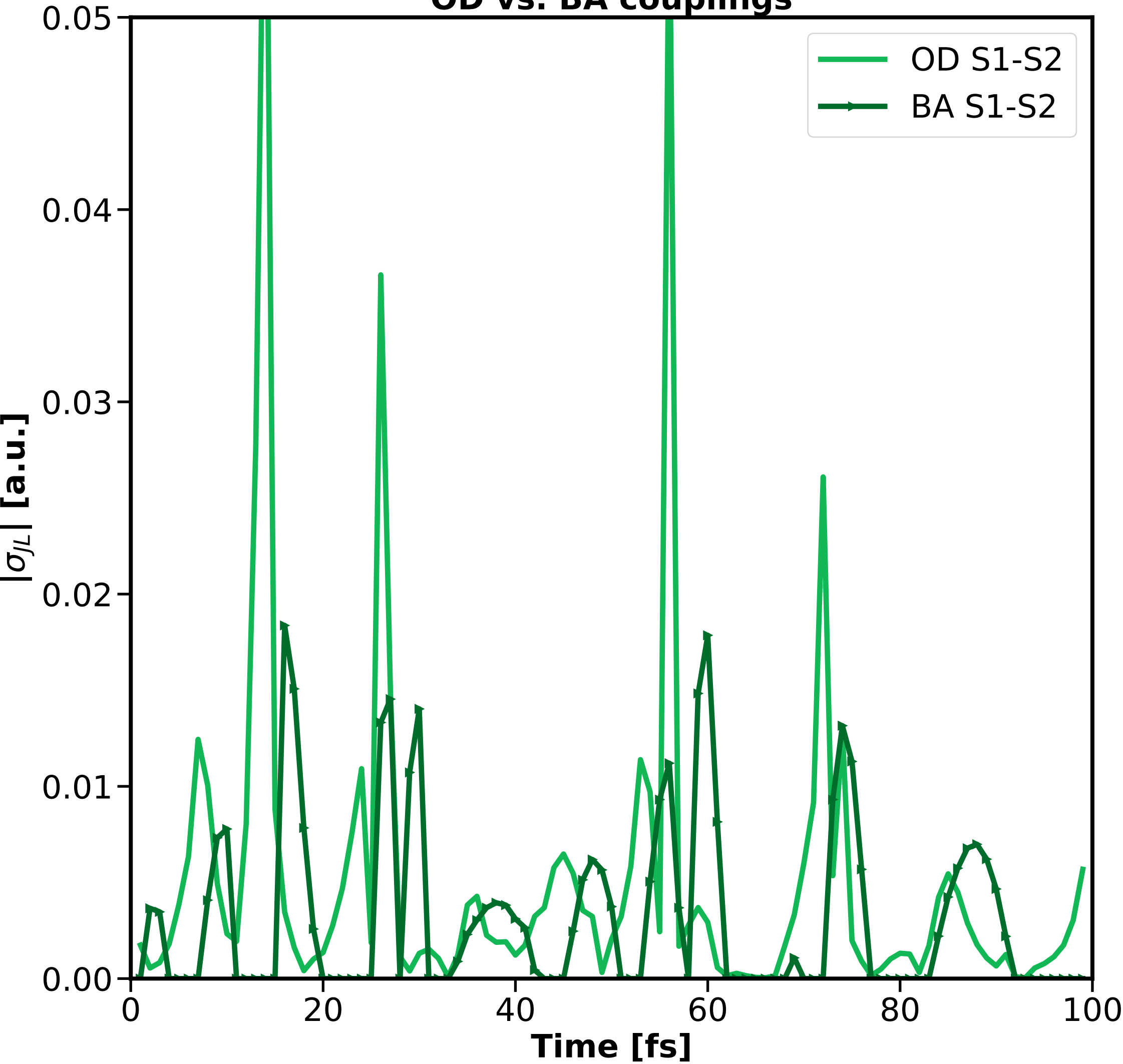

Supplement: Supplementary file 2 [file ct5c01082_si_002.zip › Supplementary_information/PICTURES/Couplings_pyrazine_crystal_OD_vs_BA_traj3_final_S1_S2.pdf]

$\delta \varepsilon = 1.0$  eV

Populations

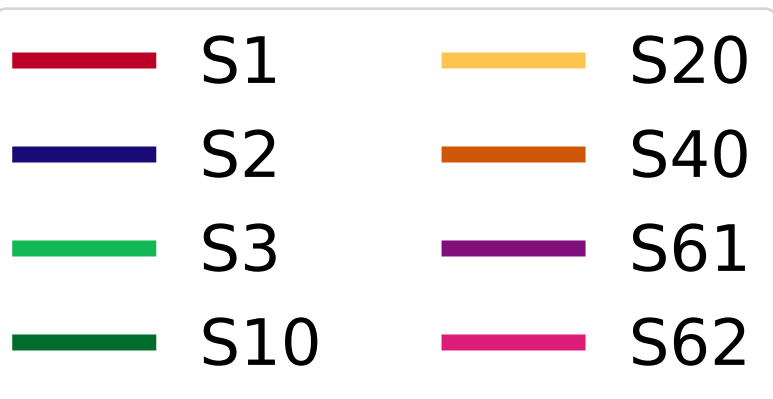

0 20 40 60 80 100

Time [fs]

Supplement: Supplementary file 2 [file ct5c01082_si_002.zip › Supplementary_information/PICTURES/population_pyrazine_pbe_crystal_molopt_dzvp_with_adiabatic_population_BA_modified_NEW_coupling_strength_of_10.pdf]

**b) CP2K / GAPW / LD with OD overlap**

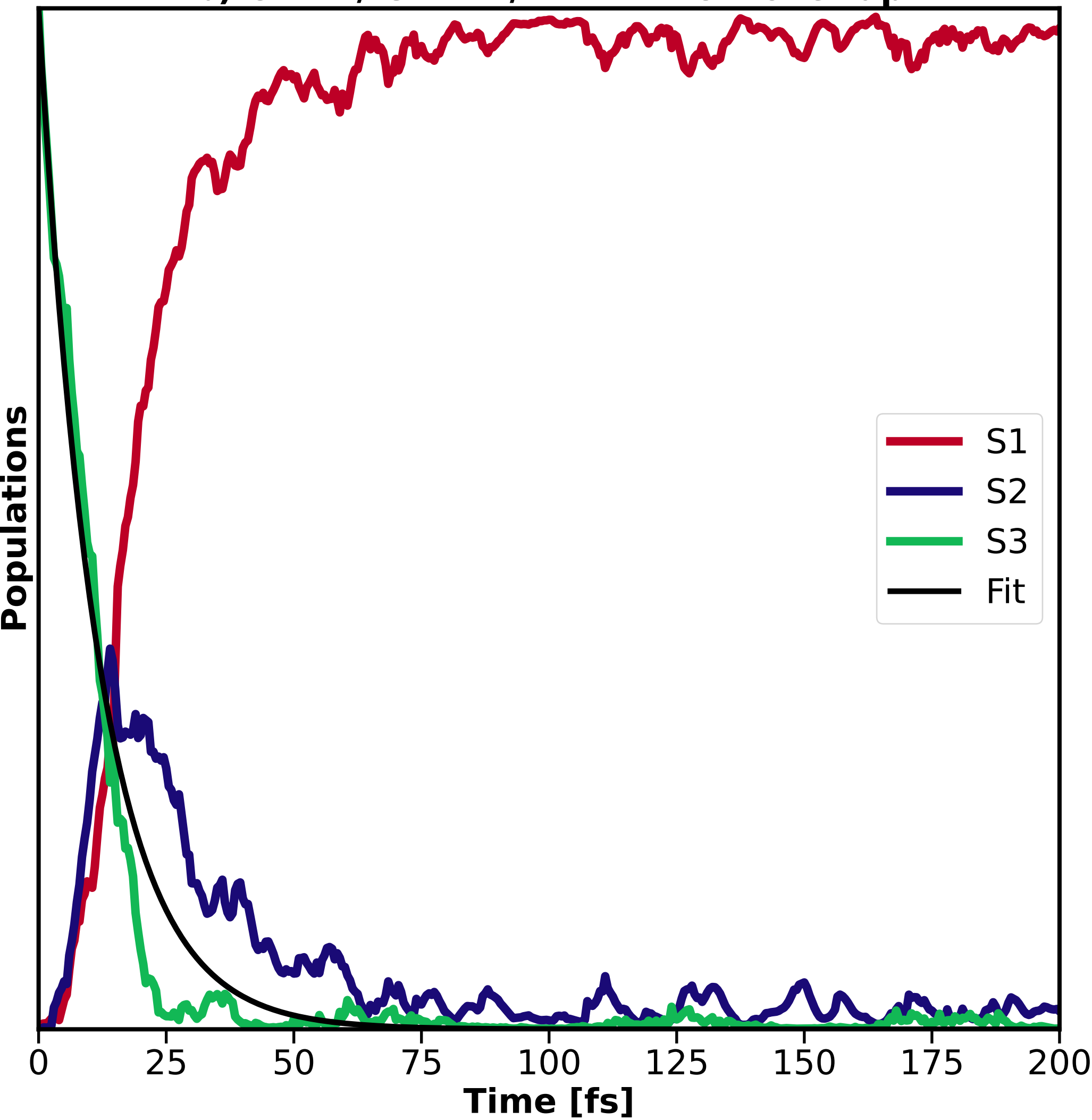

Supplement: Supplementary file 2 [file ct5c01082_si_002.zip › Supplementary_information/PICTURES/population_pyrazine_b3lyp_def2tzvp_with_adiabatic_population_NEW_LDOD_overlap_fit_2.pdf]

$\delta \varepsilon = 0.2 \text{ eV}$

Populations

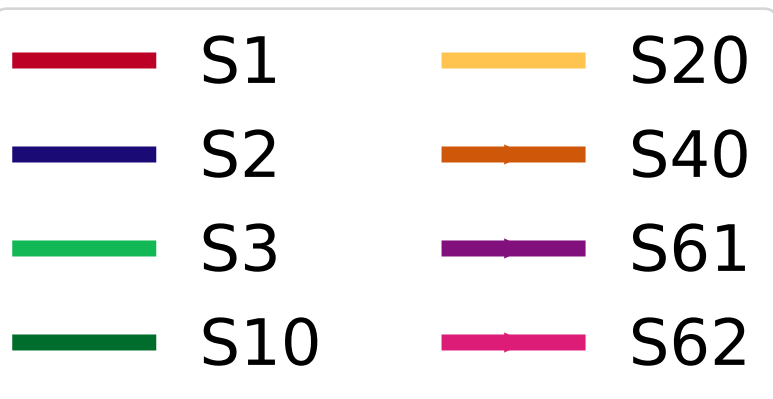

0 20 40 60 80 100

Time [fs]

Supplement: Supplementary file 2 [file ct5c01082_si_002.zip › Supplementary_information/PICTURES/population_pyrazine_pbe_crystal_molopt_dzvp_with_adiabatic_population_BA_modified_NEW_coupling_strength_of_02.pdf]

c) CP2K / sTDA / OD couplings

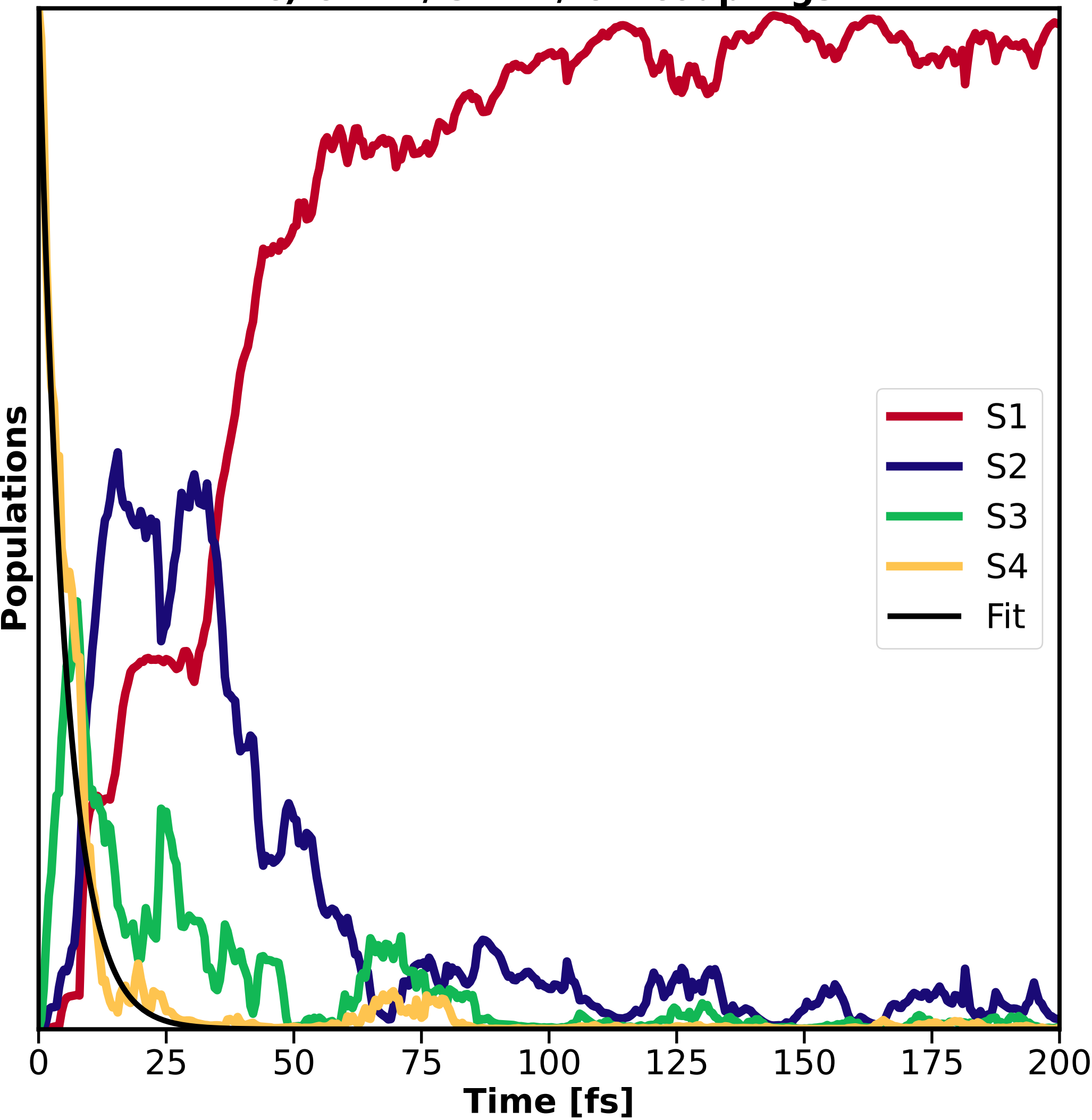

Supplement: Supplementary file 2 [file ct5c01082_si_002.zip › Supplementary_information/PICTURES/population_pyrazine_pbe_molopt_tzvp_with_adiabatic_population_DZVP_sTDA_OD_NEWcorrect_from_state_4_fit_2.pdf]

$\delta \varepsilon = 0.05 \text{ eV}$

Populations

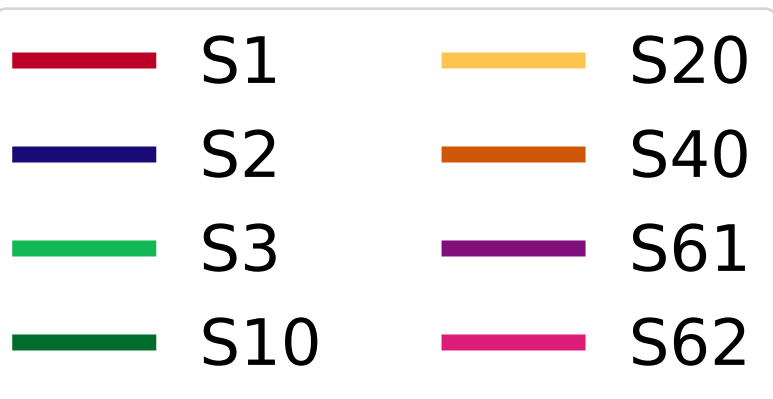

0 20 40 60 80 100

Time [fs]

Supplement: Supplementary file 2 [file ct5c01082_si_002.zip › Supplementary_information/PICTURES/population_pyrazine_pbe_crystal_molopt_dzvp_with_adiabatic_population_BA_modified_NEW_coupling_strength_of_005.pdf]

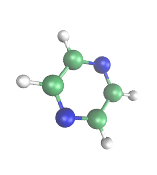

Supplement: Supplementary file 2 [file ct5c01082_si_002.zip › Supplementary_information/PICTURES/Pyrazine_visualization.png]

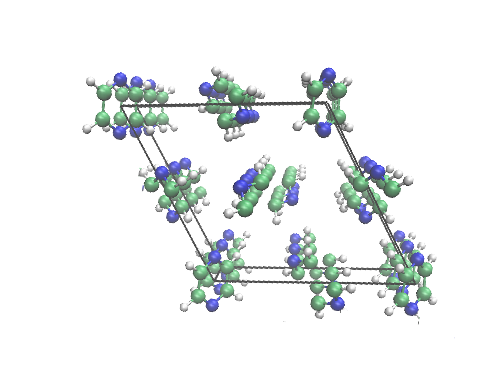

Supplement: Supplementary file 2 [file ct5c01082_si_002.zip › Supplementary_information/PICTURES/Pyrazine_crystal_visualization.png]

**OD vs. BA couplings**

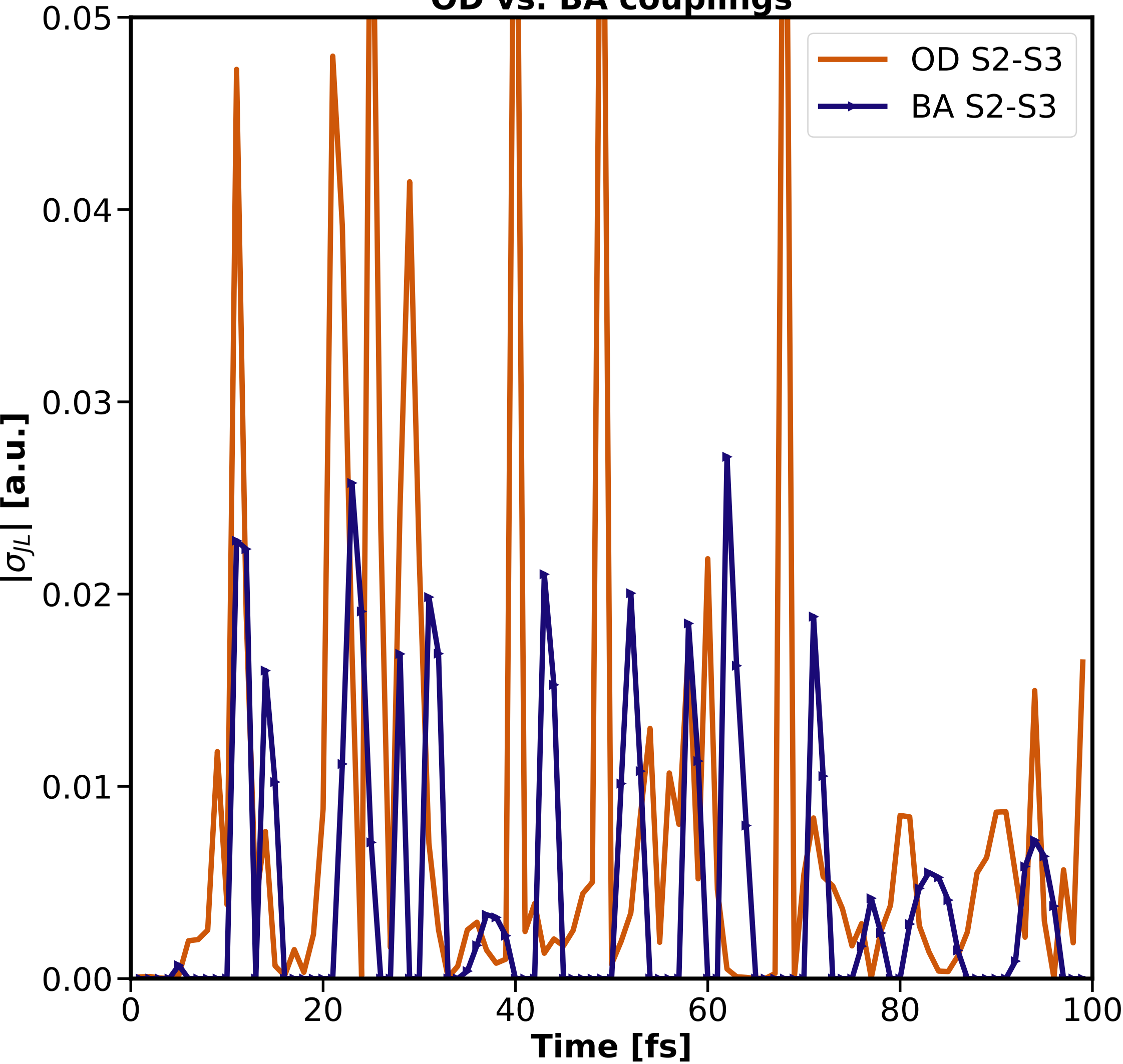

Supplement: Supplementary file 2 [file ct5c01082_si_002.zip › Supplementary_information/PICTURES/Couplings_pyrazine_crystal_OD_vs_BA_traj3_final_S2_S3.pdf]
